# Target Identification of a Class of Pyrazolone Protein Aggregation Inhibitor Therapeutics for Amyotrophic Lateral Sclerosis

**DOI:** 10.1021/acscentsci.3c00213

**Published:** 2023-12-20

**Authors:** Pathum M. Weerawarna, Isaac T. Schiefer, Pedro Soares, Susan Fox, Richard I. Morimoto, Rafael D. Melani, Neil L. Kelleher, Chi-Hao Luan, Richard B. Silverman

**Affiliations:** † Department of Chemistry, Chemistry of Life Processes Institute, Center for Developmental Therapeutics, Northwestern University, Evanston, Illinois 60208, United States; ‡ Department of Molecular Biosciences, Northwestern University, Evanston, Illinois 60208, United States; § Department of Chemistry and Proteomics Center of Excellence, Northwestern University, Evanston, Illinois 60208, United States; ∥ Department of Pharmacology, Feinberg School of Medicine, Northwestern University, Chicago, Illinois 60611, United States; @ Proteomics Center of Excellence, Northwestern University, Evanston, Illinois 60208, United States; # High Throughput Analysis Laboratory, Chemistry of Life Processes Institute, and Department of Molecular Biosciences, Northwestern University, Evanston, Illinois 60208, United States

## Abstract

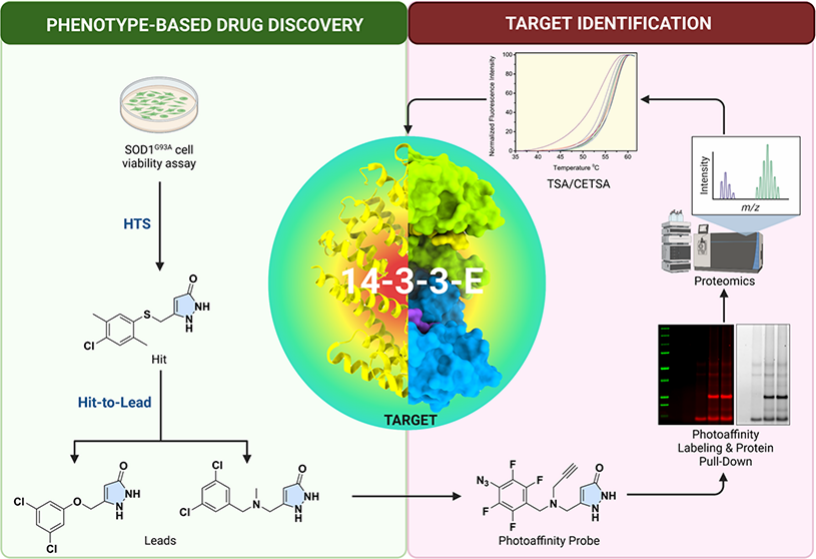

Amyotrophic lateral sclerosis (ALS) is a fatal neurodegenerative disease with no cure, and current treatment options are very limited. Previously, we performed a high-throughput screen to identify small molecules that inhibit protein aggregation caused by a mutation in the gene that encodes superoxide dismutase 1 (SOD1), which is responsible for about 25% of familial ALS. This resulted in three hit series of compounds that were optimized over several years to give three compounds that were highly active in a mutant SOD1 ALS model. Here we identify the target of two of the active compounds (**6** and **7**) with the use of photoaffinity labeling, chemical biology reporters, affinity purification, proteomic analysis, and fluorescent/cellular thermal shift assays. Evidence is provided to demonstrate that these two pyrazolone compounds directly interact with 14-3-3-E and 14-3-3-Q isoforms, which have chaperone activity and are known to interact with mutant SOD1^G93A^ aggregates and become insoluble in the subcellular JUNQ compartment, leading to apoptosis. Because protein aggregation is the hallmark of all neurodegenerative diseases, knowledge of the target compounds that inhibit protein aggregation allows for the design of more effective molecules for the treatment of ALS and possibly other neurodegenerative diseases.

## Abstract 2

14-3-3-E/Q was identified as the target of a pyrazolone molecule that extended the life of an ALS mouse model, employing a photolabile analogue, protein pulldown techniques, proteomics, and fluorescent/cellular thermal shift assays.

## Introduction

Amyotrophic lateral sclerosis (ALS) is a progressive and fatal neurodegenerative disease that is characterized by the degeneration of motor neurons in the central nervous system.^1,2^ There are two forms of ALS, sporadic ALS (SALS) and familial ALS (FALS). SALS accounts for 90–95% of the cases and has no genetically inherited attribute, while FALS accounts for the remaining 5–10% of cases that are transmitted in families, mostly as a dominant trait.^3−5^ Although the causes and precise molecular mechanisms of the motor neuronal death in ALS are unresolved, mutations in certain genes such as superoxide dismutase (SOD1), fused in sarcoma (FUS), C9*orf*72, TAR-DNA binding protein 43 (TDP-43), optineurin (OPTN), and ubiquilin-2 (UBIQL2) are known to play a significant role in both sporadic and familial ALS.^6−10^ The discovery of the dominant mutations in the SOD1 gene, which encodes the abundant cytoplasmic enzyme Cu–Zn superoxide dismutase 1, and its toxicity in FALS led to the broadening of our understanding of ALS pathology.

Mutations in SOD1 are responsible for 20% of the FALS cases whose clinical and pathological features are identical to those in SALS.^11^ There are more than 170 known ALS-causing mutations found in SOD1, and some retain their dismutase activity partially or entirely, demonstrating that ALS neurodegeneration is caused by the toxic properties of the mutant SOD1 independent of its activity.^12^ The findings from cellular and animal models expressing mutant forms of SOD1 have suggested that the primary cause of the pathogenesis and neurodegeneration is the acquired proteotoxicity from mutant SOD1 misfolding and aggregation rather than loss of function of the mutated enzyme.^13−16^ The importance of the glial cells in neurodegeneration has been demonstrated by silencing expression of mutant SOD1 in these cells.^17^ Both mutant and wild-type SOD1 interact with Ras-related C3 botulinum toxin substrate 1 (Rac1) in microglial cells, an activator of NADPH oxidase, which mediates the production of superoxide to kill pathogens. Whereas wild-type SOD1 is involved in the normal regulatory mechanism of superoxide control, mutant SOD1 strongly binds to Rac1, locking it into an active form, which elevates the level of extracellular superoxide and enhancing motor neuron damage.^15^ Microglial cells are known to cause neuroinflammation in ALS.^17,18^ Mutant SOD1, but not wild-type SOD1, inhibits Derlin-1, an endoplasmic reticulum (ER)-associated protein degradation (ERAD) component responsible for degradation of misfolded proteins, which leads to ER stress-activated apoptosis signal-regulating kinase 1 (ASK1)-dependent cell death, crucial for disease progression in FALS.^19^ Furthermore, the loss of excitatory amino acid transporter 2 (EAAT2) in astrocytes has been observed in mutant SOD1 expressing rodent models, which results in the failure to rapidly clear glutamate from the synapse, causing repetitive firing of action potentials in motor neurons. This ultimately leads to the ER and mitochondrial stress due to an increase in calcium influx.^20,21^ Mutant SOD1 has been shown to impair the expression of monocarboxylate transporter 1 (MCT1) in oligodendrocytes, which delivers the energy metabolite lactate to the axon, thereby causing a reduced supply of energy to the motor neuron.^22^ Overall, these findings suggest that ALS propagates through a noncell autonomous fashion rather than damage to neurons alone. In fact, most of the above pathophysiological features, including aberrant protein aggregation, glutamate excitotoxicity, impaired mitochondrial function, and oxidative stress, serve as possible targets for developing new therapeutics for ALS.

**Figure 1:**
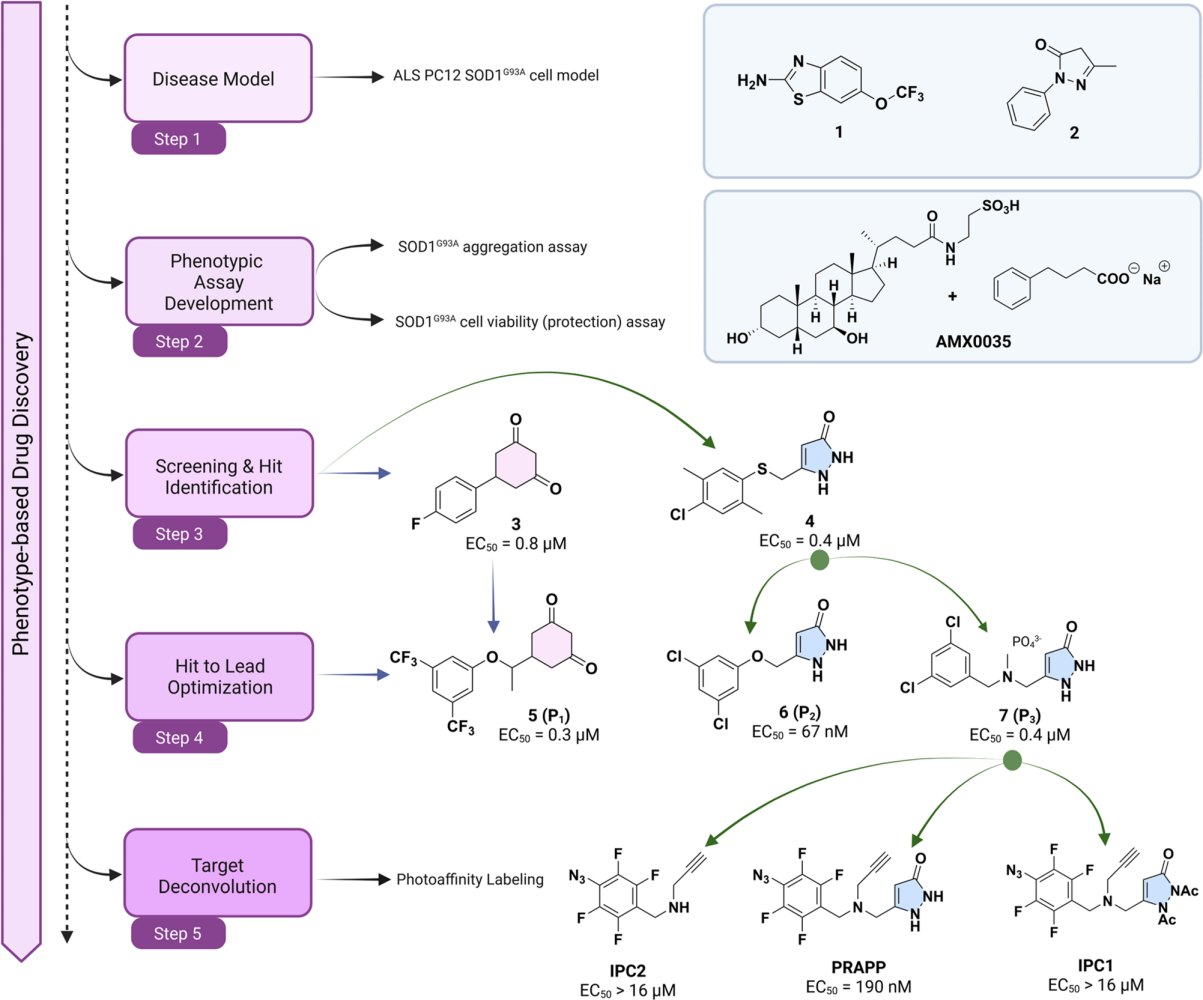
Implementation of a phenotype-based drug discovery approach to discover new ALS therapeutics and the chemical structures of riluzole, edaravone, and AMX0035.

To date, there are only three approved drugs to treat ALS, and each of these only weakly delays neurodegeneration. Riluzole (Figure 1 compound **1**) was the first drug to be approved in the U.S. for ALS, which is thought to decrease the neurotoxic effect of glutamate. A drug called edaravone (Figure 1, compound **2**), whose mechanism of action is unknown, was approved by the FDA but might act as a free-radical scavenger. Most recently, AMX0035 was approved for the treatment of ALS and is undergoing another Phase III clinical trial to assess the primary efficacy in people living with ALS globally. Some of the drugs tested for ALS were aimed at enhancing autophagy and glutamate-mediated excitotoxicity.^23^

With this information in hand, we implemented a phenotypic drug discovery approach to identify a novel therapeutic for treating ALS. The study employed a rat PC12 cell-based assay that expresses SOD1^G93A^ mutation for screening a chemical library of more than 50,000 compounds to identify promising hits (Figure 1 compounds **3** and **4**). The hits are based on attenuating proteasome inhibitor MG-132-induced SOD1^G93A^ cellular toxicity. Hit-to-lead optimization of the arylsulfanyl pyrazolone (**4**) and cyclohexane-1,3-dione (**3**) scaffolds resulted in highly potent compounds with better ADMET properties than those of the hits (Figure 1 compounds **5**, **6**, and **7**). Further studies with pyrazolone compound **6** in transgenic mice were shown to increase the median survival rate by 13%, similar to the clinical standard of care for riluzole, supporting its potential as a therapeutic candidate for ALS. Initial mechanism of action studies of these pyrazolone compounds have revealed that compounds may act as proteasome activators and do not produce a heat shock response.^24^

The final phase of the phenotype-based drug discovery process is target deconvolution (identification). There are several target identification approaches including direct biochemical methods, genetic interactions, and computational inference. These methods can be used individually or in combination to characterize on- or off-targets associated with a drug. We decided to implement direct biochemical methods to identify the target(s) of the pyrazolone lead compounds as potential ALS therapeutics. There are several direct biochemical techniques, such as activity-based protein profiling (ABPP), affinity purification, cellular thermal shift assay (CETSA), and photoaffinity labeling (PAL) techniques, that can be utilized for target identification.

Here we report a combination of PAL studies and CETSA experiments, which include the design and synthesis of a novel pyrazolone-based PAL probe for protein pull-down and proteomic identification of the target(s) as well as competition studies with the pyrazolone and cyclohexanedione compounds for target confirmation. Furthermore, we conducted fluorescence thermal shift assays (FTSAs) to quantify and confirm direct binding between the identified targets and the pyrazolone lead compounds. Finally, to robustly confirm the identified target and validate its drug–target engagement within the cellular context, we employed cellular thermal shift assays (CETSA).

## Results and Discussion

### Design and Synthesis of Pyrazolone-based Photoaffinity Labeling (PAL) Probes

Photoaffinity labeling (PAL) makes it possible to covalently modify binding partner proteins by using transient UV irradiation. Numerous PAL strategies have been described.^25^ Generally, PAL probes are designed to have the following characteristics for use in live cell culture: 1) similar structure and efficacy to a validated lead; 2) an inducible reactive functionality for labeling interacting proteins; 3) appropriate physiochemical properties to allow membrane permeation and aqueous solubility; and 4) a conjugation site for modification with chemical biology reporters. The desire to incorporate these properties, and a wealth of SAR data from our previous investigations made it possible to predict a priori which chemical probe would be suitable for our application. We chose to incorporate an aromatic photoreactive moiety in place of the terminal 3,5-dichlorophenyl group of **7**, an alkynyl conjugation site in place of the methyl group of **7**, and an active compound with improved solubility (as the HCl salt) compared to that of **5** or **6** (Figure 1). The photolabile 4-azido-2,3,5,6-tetrafluorophenyl group was chosen because this moiety indiscriminately inserts into any type of residue, including chemically “inert” aliphatic C–H bonds, which makes this moiety applicable when little information on binding residues is available.

**Figure 2:**
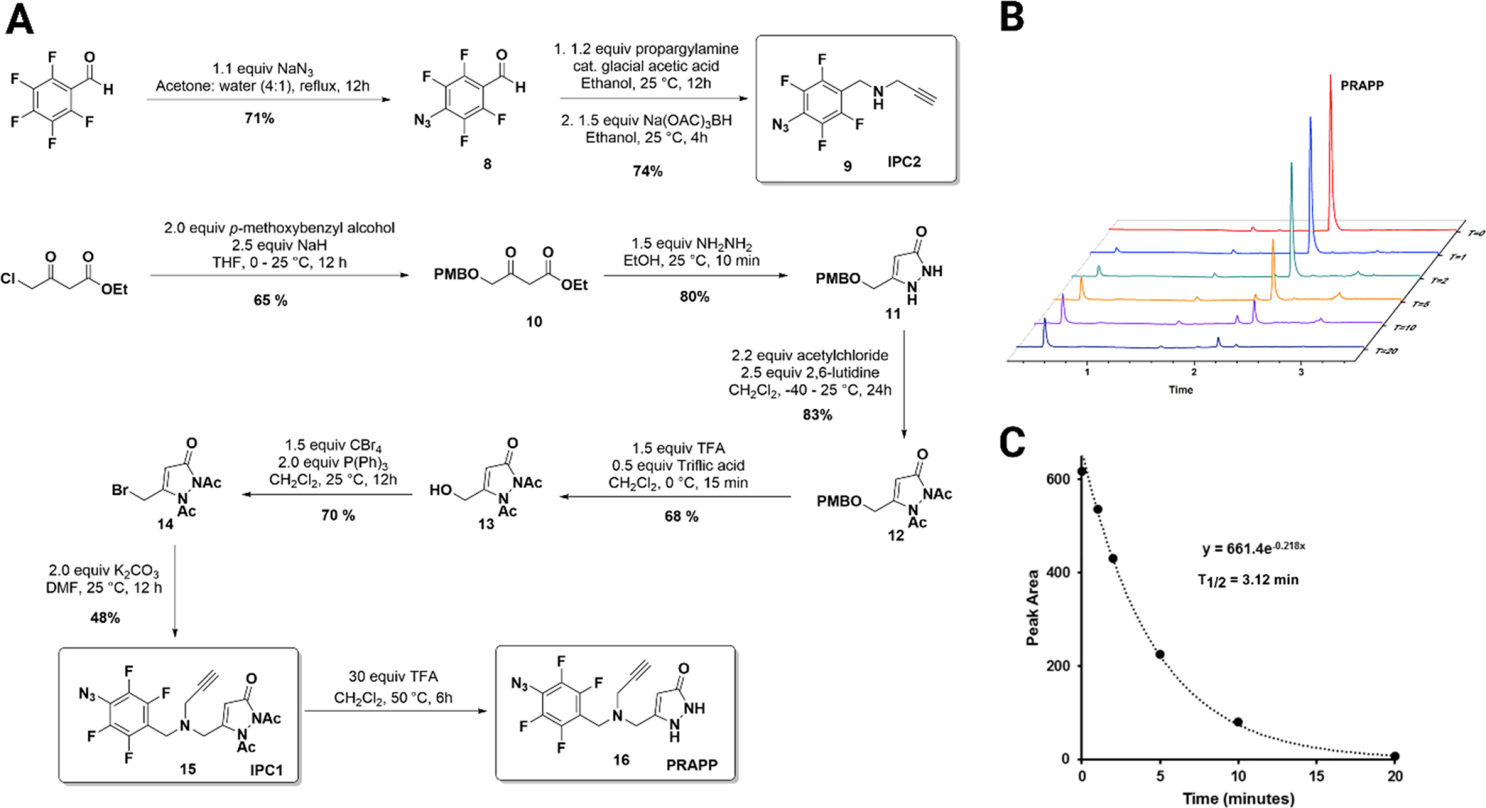
Synthesis and photolysis characterization of pyrazolone PAL probes. A) Synthetic scheme for the pyrazolone PAL probes; B) HPLC traces of the photoactivation of the PRAPP after various periods of irradiation at 312 nm; C) kinetics of the PRAPP photoactivation.

Synthesis of the desired **p**hoto**r**eactive **a**lkynyl **p**yrazolone **p**robe (abbreviated as **PRAPP**, Figure 1) was problematic and required a synthetic route different from that used to synthesize the parent molecule (P_3_), although employing common synthetic transformations (Figure 2A). Pentafluorobenzaldehyde was subjected to nucleophilic aromatic substitution with sodium azide followed by reductive amination with propargylamine to obtain intermediate **9**, which was also used as **i**nactive **p**hotoreactive **c**ontrol **2** (IPC2). Ethyl 4-chloroacetoacetate was allowed to react with *p*-methoxybenzyl (PMB) alcohol, followed by reaction with hydrazine, to obtain pyrazolone intermediate **11**, which was subjected to diacetyl protection before PMB deprotection to yield the corresponding alcohol (**13**). Functional group conversion of intermediate **13** to the corresponding bromide (**14**), followed by coupling with IPC2 resulted in intermediate **15**, which also served as **i**nactive **p**hotoreactive **c**ontrol 1 (IPC1). IPC1 is highly light-sensitive and unstable (it was stable enough to use as a control for a short period of time), which was subsequently deprotected with TFA at 50 °C to yield **16** (the **PRAPP**).

The PRAPP photolysis was monitored by following a 20 min irradiation with 312 nm UV light, which led to complete consumption of the PRAPP based on HPLC analysis (photolysis half-life 3.1 min, Figure 2B and C). The PRAPP was twice as potent as the parent molecule (**7**), having an EC_50_ of 190 nM to ameliorate MG132-induced protein aggregation and cellular toxicity in PC12 SOD1^G93A^ cells in a manner similar to that of the validated lead pyrazolones. IPC1 and IPC2 had negligible activities (EC_50_ > 16 μM).

### Covalent Modification of the Target in PC12 SOD1^G93A^ Cells by the PRAPP

**Figure 3:**
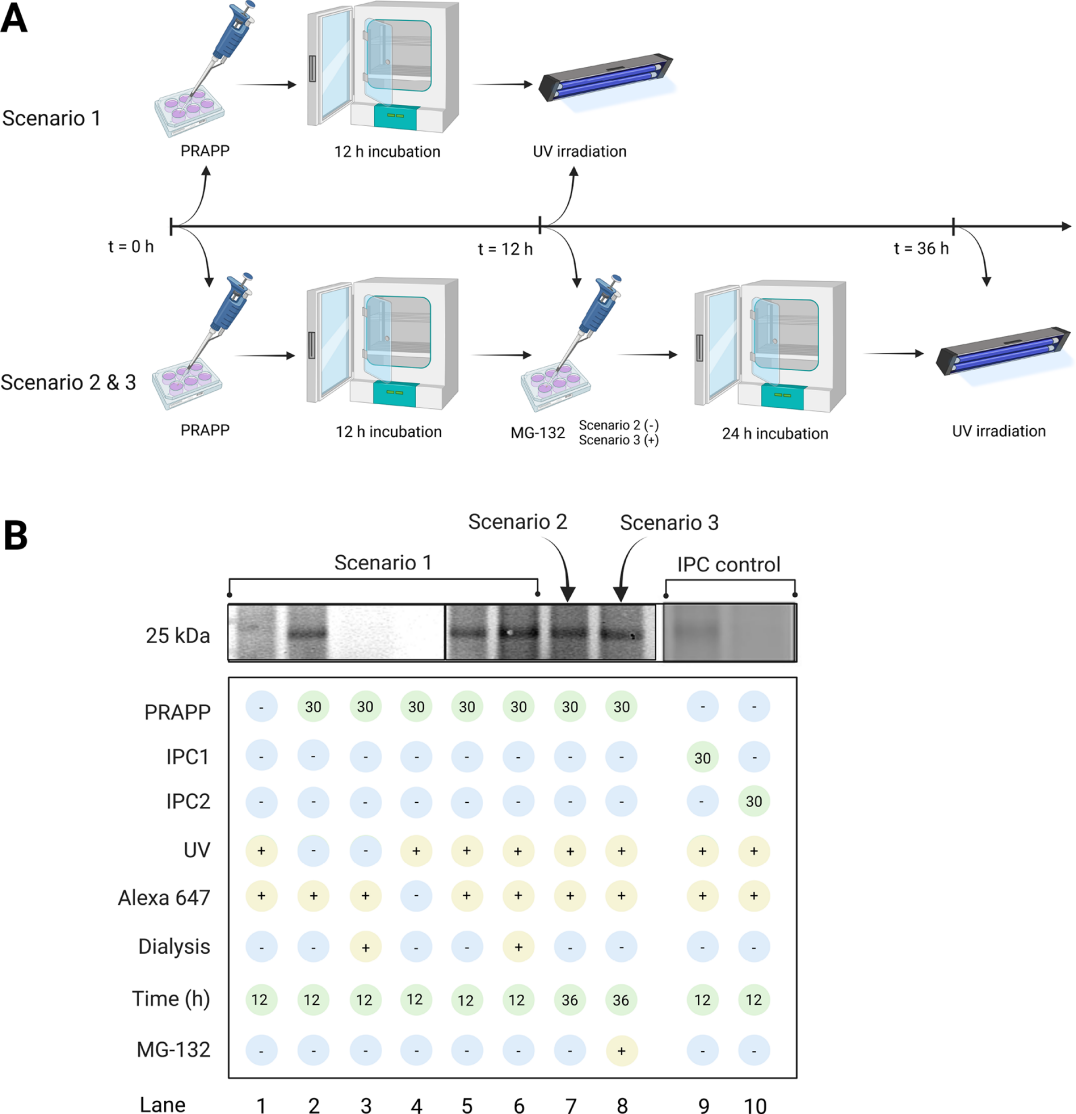
(A) PRAPP treatment scenarios and (B) covalent modification of the target by the PRAPP with different control experiments (not added is blue; added is yellow). The compound concentrations are in μM. Alexa 647 is an azido-Alexa 647. The 25 kDa covalently modified protein band was identified from this experiment. The figure was created with BioRender.

With the active photoaffinity probe and appropriate controls in hand, we next performed live cell PAL experiments to detect covalent target modification by the PRAPP. Three live cell treatment scenarios are shown in Figure 3A. Scenario 1 involved a 12 h incubation of cells with the PRAPP in the absence of MG-132 followed by subsequent UV irradiation at 312 nm for 15 min and lysis with modified RIPA buffer to collect the protein lysate. Scenarios 2 and 3 were designed to mimic the original neuroprotective assay conditions, where cells were first incubated with the PRAPP for 12 h followed by the addition of PBS (scenario 2) or MG-132 (scenario 3) and further incubated for 24 h before UV irradiation and lysis.

For the initial experiment, a 30 μM concentration of PRAPP was used, and the protein lysates were normalized to 1 mg/mL via the Bradford assay. After incubation and irradiation, the resulting protein concentrates were treated with fluorescent azido-Alexa-647, and Cu(I)-catalyzed “click chemistry” was used to conjugate the dye with the alkyne group of the PRAPP, thereby allowing visualization of covalently bound proteins after SDS-PAGE (Figure S1. Alexa-647 was used for the fluoroconjugation dye to avoid cross-contamination by the endogenous YFP fluorescence signal (from a YFP-SOD1^G93A^ conjugate)). Nonspecific labeling was a concern during methodology conceptualization, and fortunately, fluorimaging revealed a prominent modified protein band at ∼25 kDa with relatively low background labeling (Figure 3B). The full gel figure corresponding to Figure 3B (Scenarios 1, 2, and 3) is provided in the Supporting Information in Figure S2. This ∼25 kDa band was detected in all three experimental scenarios, indicating that the target was there regardless of the incubation time or the presence or absence of MG-132. Interestingly, the 25 kDa band was detected in one of the controls where cells were not subjected to UV irradiation, Figure 3B (lane 2), suggesting either 1) a strong noncovalent interaction between the PRAPP and the target protein or 2) covalent modification by the PRAPP based on its inherent reactivity in the absence of UV light. The latter interpretation was disproved based on band disappearance upon dialysis overnight, demonstrating the noncovalent nature of the initial interaction and the high affinity of PRAPP toward the target.

**Figure 4:**
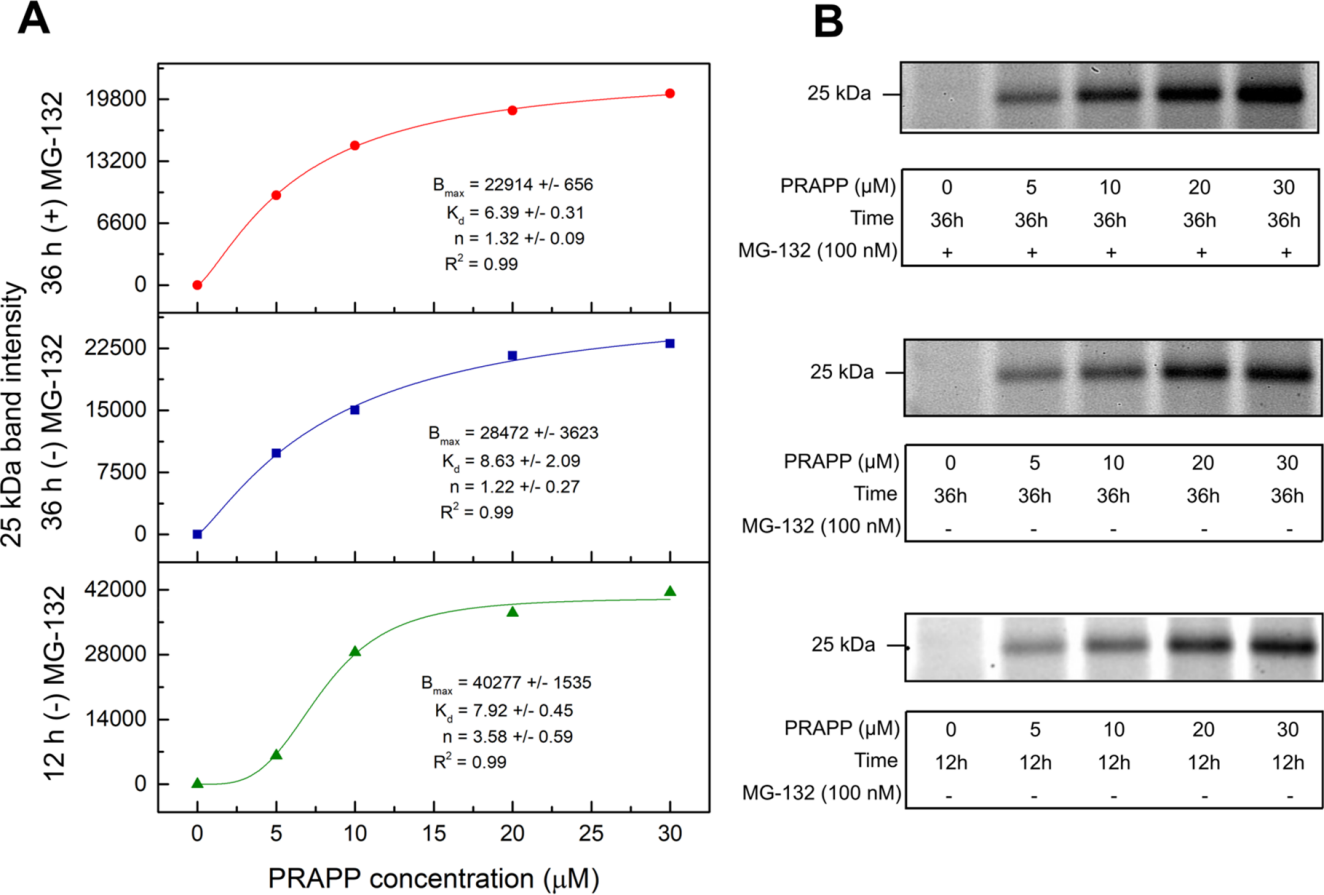
Concentration-dependent covalent modification of the 25 kDa band by the PRAPP. A) Quantified relative band intensities (*n* = 1). The plots were fitted using the Hill equation. B) In-gel fluorescence.

Further experimentation revealed a concentration-dependent increase in fluorescence intensity of the 25 kDa band under all three treatment scenarios (Figure 4A and B). The correlation between the 25 kDa band intensity and the PRAPP concentration can be described according to the Hill equation, indicating saturation binding kinetics around 30 μM of the PRAPP. The above observation suggests that the 25 kDa protein target in PC12 cells is indeed the limiting factor of the binding event, and the amount does not change as a response to an increasing concentration of the PRAPP. In addition to the PRAPP concentration, the abundance of the 25 kDa protein target in PC12 cells might depend on other factors, including incubation time and MG-132 (100 nM). As shown in Supporting Figure S3A, there is a significant intensity difference in the 25 kDa protein band between scenarios 2 and 3 (Figure 3A and B), indicating an increase in the abundance of the targeted protein as a response to MG-132 induced cellular stress.

The intensity difference between MG-132 treated and untreated samples is prominent at a 10 μM concentration of PRAPP, which gradually decreases with increasing PRAPP concentration. This observation further indicates that the PRAPP concentration range used in this experiment was close to saturation kinetics (a plateau in the Hill plot indicates that the target is saturating at higher concentrations). In a separate experiment to assess the competition of MG-132 with the PRAPP, it was revealed that the intensity of the 25 kDa band increased with MG-132 concentration in SOD1^G93A^ PC12 cells (Supporting Figure S3B). Inhibition of the proteasome by MG-132 inhibits either the degradation of the 25 kDa protein(s) or, as a response to increased SOD1 aggregation, the cell might express more of the 25 kDa target protein.

### Target Validation by Competition Experiments

Competition experiments with optimized compounds P_2_ and P_3_ (Figure 1) were performed to validate the authenticity of the 25 kDa protein band as a pyrazolone target protein. The disappearance of the 25 kDa band would be expected to occur with increasing concentrations of the competitor if this protein was the target.

**Figure 5:**
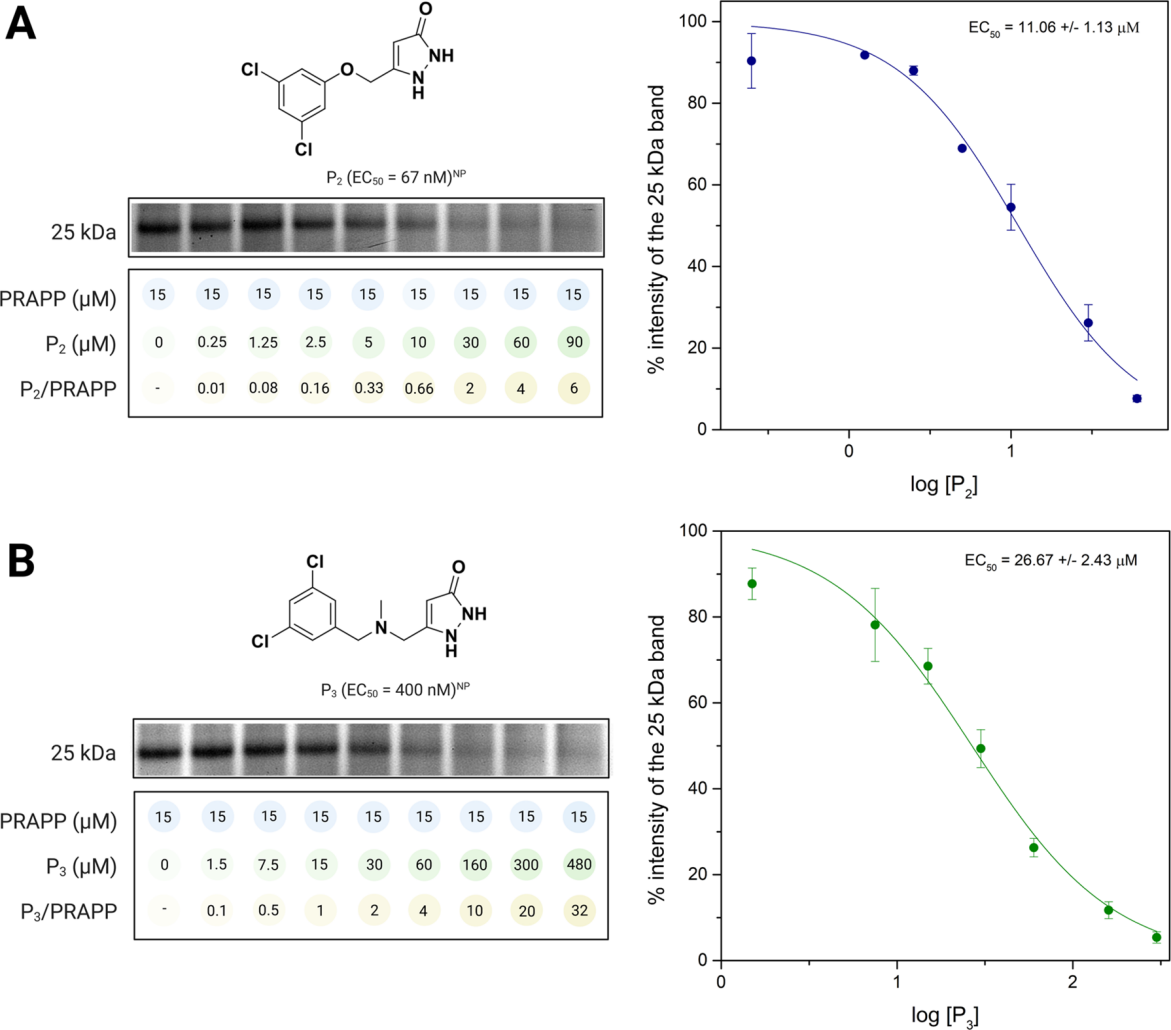
In-gel fluorescence of the competition studies of the PRAPP with (A) P_2_ and (B) P_3_; Data are presented as means ± SD from *n* = 2 independent experiments; NP = neuroprotection.

According to the results in Figure 5, pyrazolone-containing compounds P_2_ and P_3_ are capable of reducing the intensity of the PRAPP modified band in an exceptionally reliable concentration-dependent manner, confirming that the 25 kDa band is a true target of pyrazolone compounds. The calculated EC_50_ values for the competition with P_2_ and P_3_ are 11.06 and 26.67 μM, respectively. In both cases, complete disappearance of the band was observed at the P_3_ to PRAPP ratio of 32 and P_2_ to PRAPP ratio of 6, respectively. In traditional PAL experiments, it is common just to use 10 to 100-fold excess competitor concentrations, where most of the time the PAL probe is equal to or more potent than the competitor.^26−28^ This is consistent with our observations using P_2_ and P_3_, where PRAPP (EC_50_ = 190 nM) is more potent than P_3_ (EC_50_ = 400 nM) but is less potent than P_2_ (EC_50_ = 67 nM). Therefore, with the more potent competitor (P_2_), only six times excess was needed to see complete disappearance of the band, but with P_3_, 32 times excess was needed. These results also provide confirmation of a 25 kDa protein being specifically modified by PRAPP in a dynamic cellular environment and endorse this protein as having a significant role in pyrazolone-mediated neuroprotection. Importantly, competition studies with cyclohexanedione lead compound P_1_ and with edaravone did not show a quantifiable concentration-dependent disappearance of the 25 kDa protein band, indicating that, despite efficacy in our model system, neither P_1_ nor edaravone (Figure 1) binds to the same target as the pyrazolone compounds (Supporting Information Figure S4). (Full gel figures for competition experiments are given in supporting information, Figures S5–S6.)

### Protein Pull-down and Proteomics Analysis of the 25 kDa Protein Band

**Figure 6:**
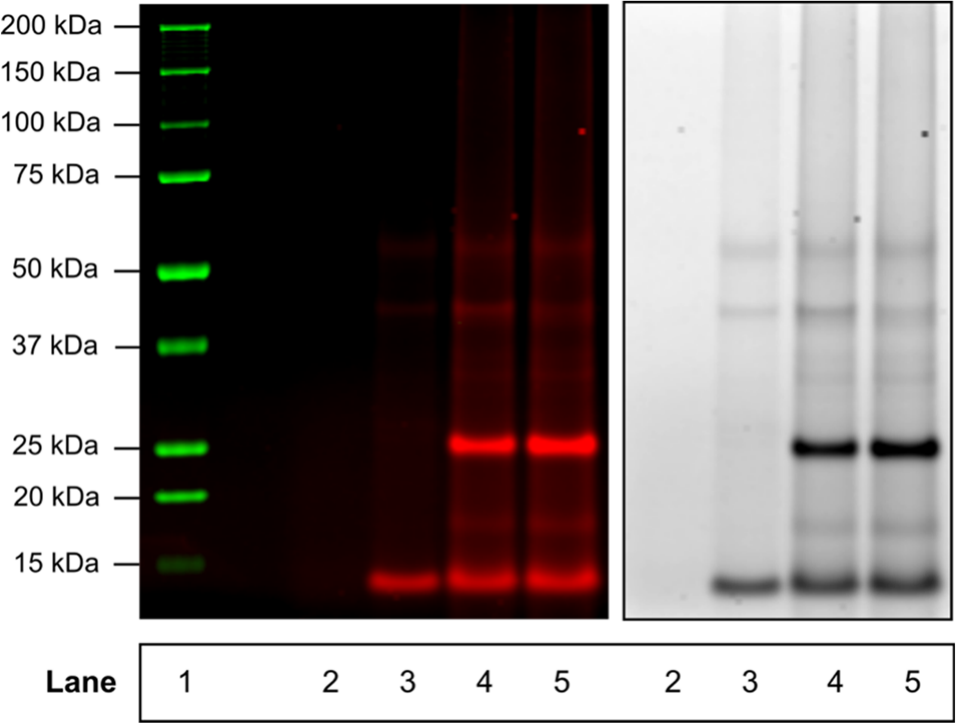
In-gel fluorescence of the protein pull-down experiment. The 25 kDa band is visible only in pull-down lanes 4 and 5, indicating the presence of the desired target. Free TAMRA-Dde-biotin bound monomeric avidin is also visible around 15 kDa. The presence of dimeric avidin is ruled out by the absence of a band around 30 kDa in the control experiments. Lane 1: Precision plus protein all blue standard; Lane 2: Control 1 (beads only + 10 min boiling in lithium dodecyl sulfate (LDS)); Lane 3: Control 2 (lysate + TAMRA-Dde-biotin azide (−Cu^2+^) + 10 min boiling in LDS); Lanes 4 and 5: Pull-down 1 and 2 (lysate + TAMRA-Dde-biotin azide + 10 min boiling in LDS). Left panel was created by merging green (ladder) and red (pull-down protein) channels using ImageJ. Single channel (pseduo-colored) image of the same gel is depicted in the right panel.

Protein pull-down with proteomics analysis was employed to identify the 25 kDa band covalently modified by the PRAPP. Concentrated protein lysates were conjugated with TAMRA-Dde-biotin azide and pulled-down using streptavidin magnetic beads. The TAMRA fluorophore allowed for visualization, and the fluorescent enrichment of the 25 kDa band from the pull-down and served as a guide in band excision for proteomics analysis (Figure S7). The dichlorodiphenyldichloroethylene (Dde) moiety was initially intended for use in the chemical digestion of the beads to minimize the release of nonspecifically bound proteins from the beads. However, chemical digestion failed, and the standard SDS boiling protocol was used. The results of the SDS-PAGE run of the pull-down protein are shown in Figure 6. As evidenced by the fluorescence image, it is clear that the pull-down experiment contains the desired 25 kDa target, which is absent in the control experiment.

In-gel digestion is a widely used technique in target identification, which involves the fluorescent-guided excision of the covalently modified band, followed by target protein identification by bottom-up proteomics under label-free conditions, based on its enrichment compared to the control sample.^29−31^ The fluorescent-guided gel band excision aids in precisely selecting the target protein from a mixture of bands, distinguishing it from nonspecific proteins, including streptavidin. On-bead digestion is another target identification technique that is typically coupled with Stable Isotope Labeling by Amino Acids in Cell Culture (SILAC) and quantitative proteomics. This technique addresses challenges such as the identification of low-abundance targets that are difficult to detect with in-gel digestion. However, we chose in-gel digestion over on-bead digestion, because our fluorescence labeling experiments indicated that the target of interest is highly abundant in the cell matrix. Proteomics analysis of the excised bands identified six potential candidates as the target protein with known chaperone function, which include five of the seven 14-3-3 protein family members (14-3-3-β/B, 14-3-3-ε/E, 14-3-3-η/H, 14-3-3-γ/G, and 14–4–3-θ/Q) and heat shock protein 27 (Hsp27) (Table S1). According to the normalized total spectral value, all candidates were enriched in the pull-down samples compared with the controls; only 14-3-3-E was absent in the control. The top three protein candidates include 14-3-3-θ, with a normalized total spectral count of 25 in the pull-down sample, followed by 14-3-3-η and Hsp27, with total spectral counts of 17 and 16, respectively. Four other proteins were identified within the 23–28 kDa range, namely, RAB5C (member of the RAS oncogene family), glutathione S-transferase, Ras-related protein Rab, and triosephosphate isomerase, but they do not have a known function in the literature related to protein aggregation. Consequently, the follow-up investigation focused on 14-3-3 proteins and Hsp27.

The 14-3-3 proteins form homo- or heterodimers that are in equilibrium with the corresponding monomers. Each monomer consists of a conserved phosphoprotein binding site that interacts with its non 14-3-3 binding partners.^32^ The isoform structures are conserved except at the areas of the interface that form intersubunit contacts. The dimeric structure is mainly stabilized by the specific salt-bridge interactions that exist between the subunits. Theoretically, homodimers of B, Z, and Q isoforms of human 14-3-3 proteins can form six intersubunit salt-bridge interactions, while G and H isoforms can only form four.^33^ The E isoform can create only two intersubunit salt-bridges in its homodimeric form, whereas additional salt-bridge interactions exist in the heterodimeric conformation. Therefore, the 14-3-3-E isoform preferentially forms heterodimers rather than homodimers in the cellular environment. The dimeric structure is essential for the scaffolding function of 14-3-3 proteins, where it brings two client proteins together to form a specific structure. Furthermore, the binding partners with two 14-3-3 binding sites rely on the dimeric structure of 14-3-3. Even though potential candidates have been identified, the protein’s smaller size and the presence of homo- and heterodimers make the target protein’s definitive identification somewhat ambivalent. Genetic manipulation is a technique that can be used to confirm the identity of the target candidates by systematically deleting the genes corresponding to each candidate and conducting photoaffinity labeling experiments. The implementation of this technique here is challenging due to the multiple potential candidates identified by proteomics, which require the generation of a series of knockout PC12 cell lines for each candidate. However, because of the commercial availability of the HAP1 Hsp27 knockout cell line (Horizon Discovery, HZGHC004733c006), we decided first to conduct photoaffinity labeling studies in HAP1 cells to determine if Hsp27 is a definitive target of the pyrazolone compounds.

### Photoaffinity Labeling Studies with HAP1 Hsp27 Knockout (KO) Cell Line

**Figure 7:**
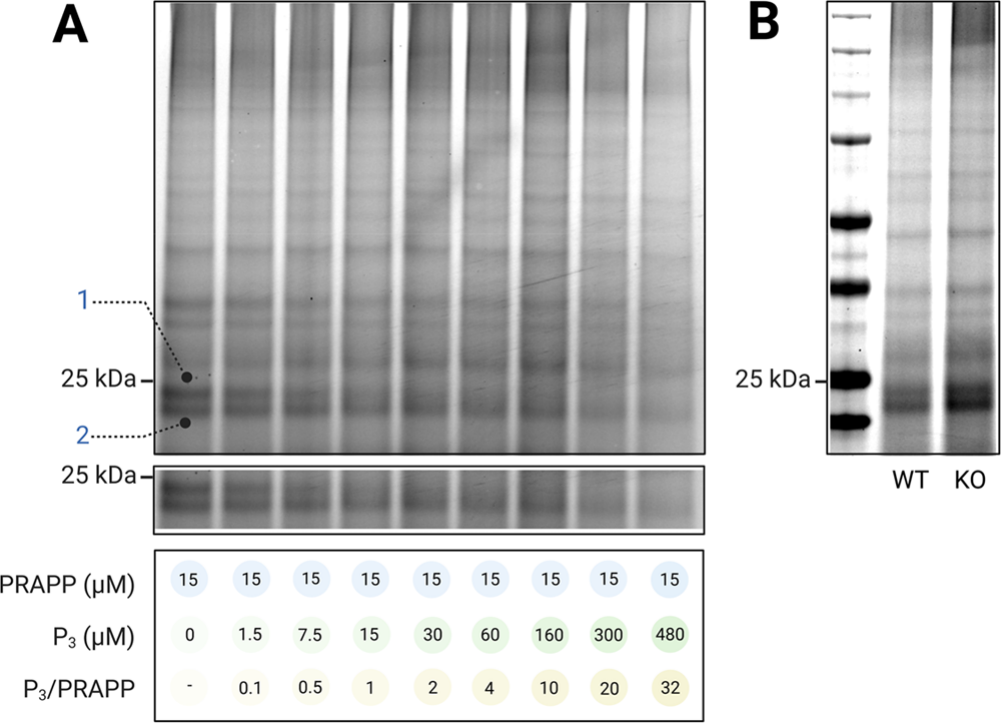
Photoaffinity Labeling Experiments with HAP1 cells. (A) In-gel fluorescence of the competition of PRAPP with P_3_ (band 1 showed a concentration-dependent disappearance with P_3_) in wild-type (WT) HAP1 cells. The nonspecific background modifications remain unchanged with increasing concentration of P_3_, further confirming the 25 kDa covalently modified band as a true target; (B) comparison of the photoaffinity labeling experiments with HAP1 wild-type (WT) cells and Hsp27 knockout (KO) cells at 15 μM PRAPP concentration.

First, we conducted photoaffinity labeling studies with wild-type (WT) HAP1 cells to verify the existence of the 25 kDa covalent modification that we observed in PC12 cells. Significant nonspecific background modifications were present in HAP1 cells compared to PC12 cells, as shown in Figure 7. There were two covalently modified bands around 25 kDa (bands 1 and 2), where only band 1 showed a concentration-dependent disappearance with P_3_ competition (Figure 7A), which confirmed the existence of the 25 kDa modified band in HAP1 cells. However, in Hsp27 KO HAP1 cells, the intensity of the 25 kDa covalently modified band remains unchanged compared to that in WT cells, thereby eliminating Hsp27 as the target of the pyrazolone compounds (Figure 7B).

Implementation of a similar experimental procedure with 14-3-3 isoforms was challenging due to the number of isoforms suggested by proteomics and the lack of readily available knockout cell lines. However, biological (functional) or physiological assays can be used to evaluate the direct binding between potential target(s) and the drug candidate to overcome this challenge. Therefore, we decided to evaluate the direct binding of P_2_ and PRAPP with isolated potential 14-3-3 protein isoform targets to confirm the proteomics findings. The lack of functional assays for 14-3-3 isoforms initially made it difficult to achieve this goal. The fluorescent thermal shift assay (FTSA) (also known as differential scanning fluorimetry (DSF)) is a widely employed technique to confirm direct protein−protein and protein−small molecule binding. Thus, we carried out FTSAs to demonstrate direct binding of the lead pyrazolone compound (P_2_) and the PRAPP with the isolated 14-3-3 protein targets in vitro.

### Fluorescent Thermal Shift Assays (FTSAs) of Isolated Protein Targets with P_2_ and PRAPP

**Figure 8:**
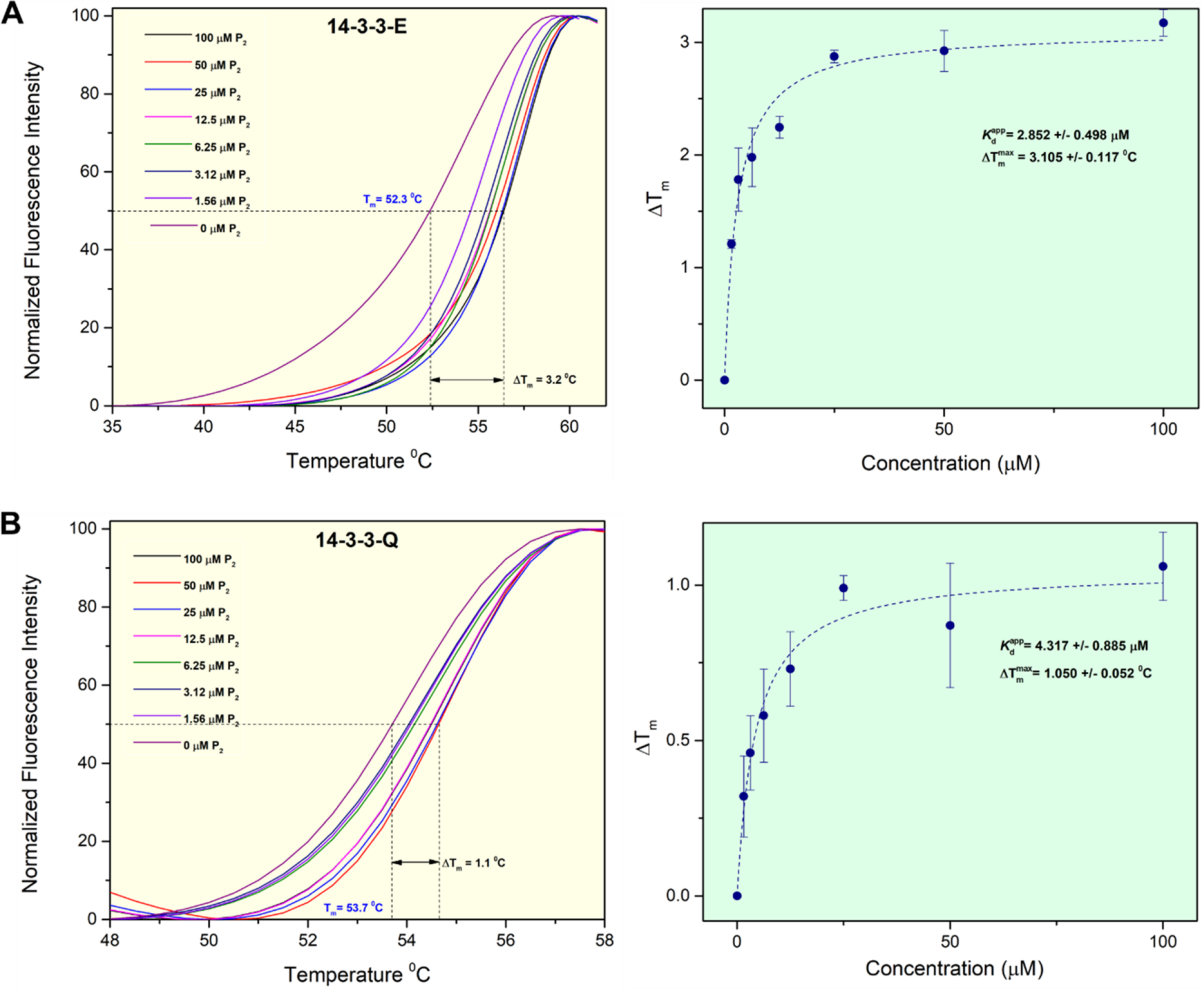
FTSA results of P_2_ with (A) 14-3-3-E and (B) 14-3-3-Q. Melting curves are shown in the left panel, and the Δ*T*_m_ vs P_2_ concentration fitting curves are shown in the right panel. Δ*T*_m_ values are presented as means ± SD from *n* = 2 independent experiments.

The direct binding of small molecules affects the protein thermal stability and shifts the melting temperature (*T*_m_). FTSAs quantify the changes in *T*_m_ using the SYPRO orange fluorescent probe. The quantum yield of the environment-sensitive dye increases significantly when binding to hydrophobic surfaces exposed when a protein unfolds. The melting temperatures of the 14-3-3 isoforms tested had *T*_m_ values around 53 °C, which is close to the values reported in the literature.^34^ However, the FTSA results indicated that only 14-3-3-E and Q isoforms showed a significant *T*_m_ shift upon incubation with P_2_ (Figure 8); no binding of P_2_ to isoforms B, H, and G was observed (Supporting Information Figure S8). The 14-3-3-E isoform demonstrated a major *T*_m_ shift (3.2 °C), indicating a stronger binding of P_2_ and stabilization, while 14-3-3-Q showed a minor *T*_m_ shift (1.1 °C). Based on these data, we estimated the apparent binding constants (*K*_d_^app^) of P_2_ with the two isoforms by fitting the Δ*T*_m_ values and P_2_ concentrations. The estimated *K*_d_^app^ values for P_2_ with 14-3-3-E and 14-3-3-Q are 2.85 and 4.32 μM, respectively. The observed major *T*_m_ shift for 14-3-3-E with P_2_ led us to continue the FTSA experiments to evaluate the binding of PRAPP and P_1_, the cyclohexanedione lead molecule, with 14-3-3-E. PRAPP showed a *T*_m_ shift of 2.5 °C, which is similar to that for P_2_, confirming the direct and robust binding of pyrazolone compounds with the 14-3-3-E isoform (Figure S9). However, P_1_ did not show a *T*_m_ shift, which is consistent with the outcome of the competition experiment (Figure S10).

Overall, the FTSA experiments revealed direct binding of pyrazolone compounds P_2_ and PRAPP with 14-3-3-E, as indicated by major *T*_m_ shifts. In addition, 14-3-3-Q also displayed a minor *T*_m_ shift with P_2_, indicating some binding interactions. These findings suggest that 14-3-3-E is the main target of the pyrazolone compounds and that 14-3-3-Q is a minor target. Even though 14-3-3 isomers B, H, and G did not show any binding with pyrazolone compounds, the presence of those isomers in the pull-down protein matrix can be attributed to 14-3-3-E forming heterodimers with isoforms B, H, and G in cells. The covalent modification of 14-3-3-E heterodimers in cells eventually pulls down B, H, and G isoforms attached to 14-3-3-E, making those proteins appear in the proteomics results. Based on this observation, it also can be concluded that the binding of pyrazolone and PRAPP does not disrupt the formation of the heterodimers if it were to bind at the interface, in which case the other isoforms would not have been pulled down.

Finally, given the major *T*_m_ shift observed for 14-3-3-E with P_2_ and PRAPP, we decided to focus on 14-3-3-E to evaluate these binding interactions in a cellular environment. As the final step in target identification, this kind of confirmation is essential for validating the identified target within the relevant cell model. The Cellular Thermal Shift Assay (CETSA) is a widely used technique for confirming cellular target engagement of drugs, employing a principle similar to that of FTSA. Therefore, we conducted CETSA experiments to evaluate the direct binding of the lead pyrazolone compound P_2_ to 14-3-3-E in vivo within the PC12^G93A^ cellular environment.

### Evaluation of P_2_ Interactions with 14-3-3-E in PC12^G93A^ Cells using Cellular Thermal Shift Assay (CETSA)

The CETSA experiment was first described by Molina et al. as a technique to monitor drug target engagement in cells and tissues, utilizing either intact cells/tissues or cell lysates.^35^ This process involves heating multiple aliquots of cell lysate to different temperatures and subsequent centrifugation to separate the soluble fraction from the precipitated proteins upon cooling. Thereafter, the presence of the target protein in the soluble fraction is quantified by Western blotting.

**Figure 9:**
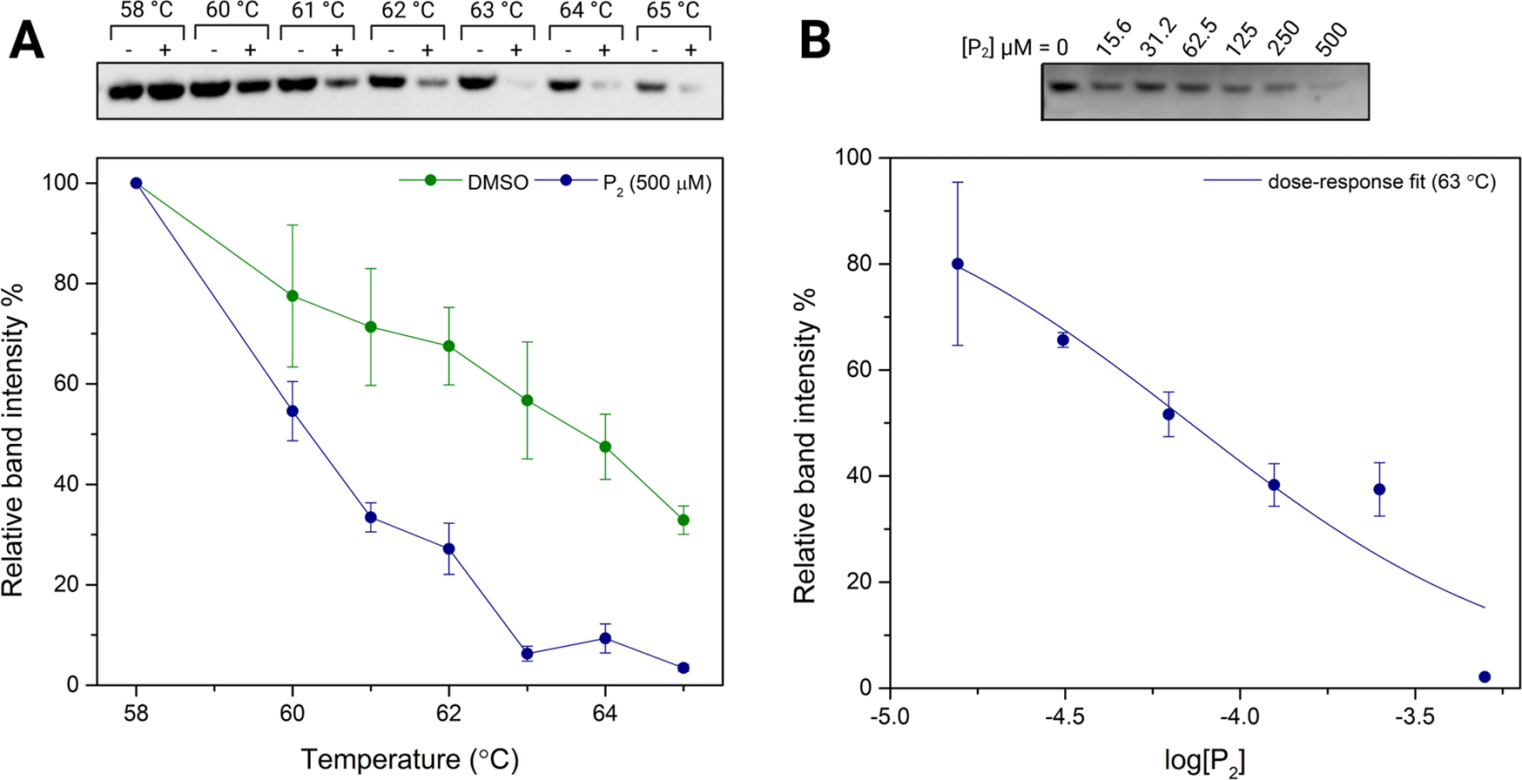
CETSA melting curve of 14-3-3-E at 500 μM P_2_ concentration (A) and isothermal dose–response (ITDR) CETSA curve of 14-3-3-E at 63 °C (B) in PC12^G93A^ cell lysate. Data are presented as means ± SEM from *n* = 3 independent experiments.

Our initial attempts to conduct the experiment in intact PC12-SOD1^G93A^ cells were unsuccessful, likely due to thermal lysis of the cells at high temperatures. Consequently, we decided to switch to the cell lysate protocol. We first conducted the experiment at a high concentration of P_2_ to determine if there was a significant thermal shift associated with 14-3-3-E in the presence of P_2_ in the cell lysate. Interestingly, a significant relative band intensity difference was observed for 14-3-3-E with P_2_ compared to the DMSO control, between 60 and 64 °C, with the maximum difference noted at 63 °C (Figure 9A). Overall, the enhanced thermal stability of 14-3-3-E in the cell lysate, compared to that of the purified protein (*T*_m_ = 52.3 °C), can be attributed to the protein–protein interactions it forms with non-14-3-3 binding partners and the heterodimer formation in the cellular environment. Such interactions further stabilize the protein, shifting its *T*_m_ to a higher value. However, in contrast to common observations in which small molecule binding increases the thermal stability of a protein, the addition of P_2_ markedly reduced the presence of 14-3-3-E in the soluble fraction, indicating a destabilization of 14-3-3-E in the cell lysate. In fact, this kind of decreased thermal stability is rare but has been reported in the literature associated with small molecules that disrupt protein–protein interactions and complex formation.^36^ Since in vitro FTSA indicated increased thermal stability and noninterruption of 14-3-3-E dimer formation in the presence of P_2_, the observed in vivo destabilization might result from the potential disruption of protein–protein interactions between 14-3-3-E and its non-14-3-3 binding partners in the cellular environment due to P_2_, thereby lowering the thermal stability. This observation is highly significant for assigning a potential function associated with the 14-3-3-E and P_2_ binding interactions in the context of ALS pathology. It can be hypothesized that the neuroprotective phenotypic outcome of P_2_ is linked to the potential disruption of interactions between 14-3-3-E and its non-14-3-3 binding partners in PC12^G93A^ cells.

Following the establishment of the thermal shift of 14-3-3-E in the presence of P_2_ at high concentrations, we conducted isothermal dose–response (ITDR) experiments to evaluate the concentration dependence of the relative band intensity of 14-3-3-E in the soluble fraction at 63 °C. As shown in Figure 9B, a clear decrease in the relative band intensity of 14-3-3-E was observed with increasing concentrations of P_2_, confirming the concentration dependence of 14-3-3-E protein stability. Interestingly, this response was reached at relatively high P_2_ concentrations, an observation also noted in the original CETSA protocol that used cell lysate compared with intact cells. For instance, drugs with nanomolar inhibitor potencies, such as raltitrexed and methotrexate, displayed a maximum response between 100–250 μM drug concentrations. The authors attributed this observation to factors such as the higher temperatures used in the experiment, potential protein binding, metabolic factors at higher lysate concentrations, and the nonequilibrium nature of protein precipitation upon unfolding, suggesting the use of the ITDR CETSA experiment as a fingerprint for target engagement across a range of drug concentrations. Overall, the CETSA experiments we conducted confirm the P_2_ interactions with 14-3-3-E in a cellular context and validate 14-3-3-E as a definitive target of the pyrazolone compounds.

### Importance of 14-3-3-E and 14-3-3-Q in ALS Pathogenicity

The role of protein aggregation as the primary causative agent in neurodegenerative diseases remains a topic of debate, with some studies suggesting that smaller soluble oligomers might be the principal toxic species rather than larger aggregates.^37^ Additionally, there are observations indicating that protein aggregates may represent a cellular protective response to sequester misfolded proteins, challenging the traditional perspective of their pathogenic role.^38^ Even though the precise mechanism of ALS pathogenesis is unclear, protein aggregation is still one of the main pathological features, which is also common in other neurodegenerative diseases, such as Alzheimer’s disease (AD), Parkinson’s disease (PD), and Huntington’s disease (HD). There are mainly two classes of aggregate-prone protein phenotypes distinguished by a cellular quality control mechanism, namely, soluble normal misfolded proteins and insoluble amyloidogenic proteins.^39^ Mammalian cells are known to localize these aggregates at distinct inclusion sites.^40,41^ Normal soluble misfolded proteins consist of ubiquitinated proteins with mutations or stress-induced damage, and they accumulate in the Juxta Nuclear Quality control compartment (JUNQ) adjacent to the nucleus. Amyloidogenic proteins form insoluble aggregates and are sequestered in Insoluble Protein Deposit-like (IPOD-like) inclusions.^39^

**Figure 10:**
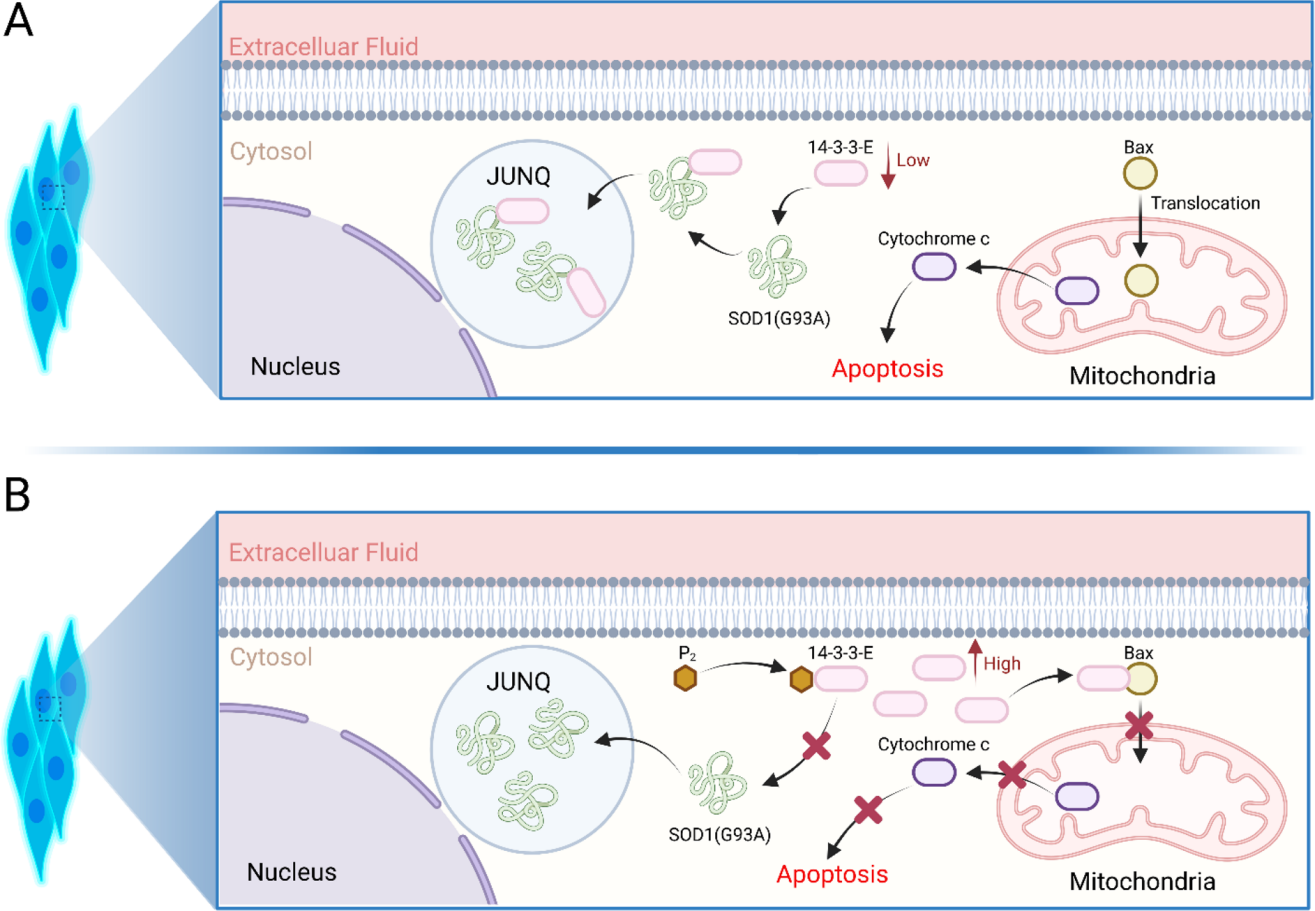
(A) Interaction of 14-3-3-E and 14-3-3-Q with SOD1^G93A^ (the figure was constructed based on the findings of ref (42)); (B) proposed hypothesis for the disruption of 14-3-3-E and 14-3-3-Q interactions with misfolded SOD1^G93A^ in the presence of pyrazolone compounds and prevention of 14-3-3 cosequestration into JUNQ. The figure was created with BioRender.

A recent study has shown that in the SOD1^G93A^ ALS mammalian cell line, aggregation of insoluble SOD1^G93A^ aggregates in JUNQ interferes with its quality control function.^39^ Furthermore, it has been demonstrated that sequestration of these SOD1^G93A^ aggregates in an insoluble inclusion (e.g., in IPOD) reduces the harmful effects of aggregation on the cell viability. Another recent independent study found that in the SOD1^G93A^ ALS transgenic mouse model, 14-3-3 proteins colocalize with mutant SOD1 aggregates, making them more insoluble in the spinal cord than in wild-type mice.^42^ The same study found that 14-3-3-E and 14-3-3-Q isoforms interact with mutant SOD1 aggregates in the JUNQ of N2a neuroblastoma cells and become insoluble. Consequently, the cytosolic abundance of 14-3-3-E and 14-3-3-Q isoforms decreases, promoting the translocation of Bax (Bcl-2-associated X protein) into the mitochondria and releasing cytochrome c into the cytosol, causing apoptosis (Figure 10A). A proposed function of 14-3-3 proteins is to inhibit apoptosis by interacting with Bad (Bcl-2-associated death promoter) or Bax, which prevents the localization of these proteins into the mitochondria. Furthermore, overexpression of 14-3-3-E and 14-3-3-Q in the same cell line dramatically decreased SOD1^G93A^-induced cytotoxicity. Based on these literature findings, we hypothesize that the pyrazolone compounds disrupt the 14-3-3-E and 14-3-3-Q interactions with misfolded SOD1^G93A^ and prevent cosequestration into JUNQ, keeping the cytosolic 14-3-3 level adequate to maintain its regular function (Figure 10B). As previously discussed, the observed decrease in the thermal stability of 14-3-3-E in the PC12^G93A^ cell lysate upon P_2_ exposure, coupled with the absence of SOD1 in the pull-down matrix, further supports our hypothesis that the pyrazolone compounds disrupt the 14-3-3-E-SOD1^G93A^ interactions. 14-3-3 proteins are also proposed to function as molecular chaperones that interact with aggregation-prone proteins or to target misfolded proteins to aggresome structures. These results strongly support our experimental finding of 14-3-3-E (in vitro/in vivo) and 14-3-3-Q (in vitro) as molecular targets of pyrazolone compounds and their functional importance in ALS pathogenesis.

## Conclusions

ALS is a progressive and fatal neurological disorder that affects neurons in the central nervous system. We launched a phenotypic drug discovery campaign to discover a novel therapeutic that inhibits protein aggregation for treating ALS. One class of compounds that we optimized was the pyrazolone, which was active in extending the life of the ALS mouse model and was shown to maintain the health of axons in upper motor neurons from a mouse with mutant SOD1 toxicity in vitro (unpublished data). As the last stage of our phenotype-based ALS drug discovery approach, we have successfully conducted direct biochemical studies to find molecular targets of the pyrazolone-based neuroprotective compounds optimized from hits in our screening campaign. First, we designed a tetrafluoroazidobenzene-containing photoaffinity probe based on neuroprotective pyrazolone compound P_3_, and from subsequent photoaffinity labeling studies, we identified a 25 kDa covalently modified protein band in the PC12 SOD1^G93A^ cell line. The covalent modification is concentration-dependent, and the competition experiments revealed that both pyrazolone compounds (P_2_ and P_3_) and the photoaffinity probe (PRAPP) compete for the same 25 kDa protein target. Subsequent protein pull-down experiments, followed by proteomics analysis of the 25 kDa modified protein band, revealed that this protein belongs to the 14-3-3 family of proteins. To confirm the identity and obtain direct binding evidence, we expressed and purified the 14-3-3 family proteins identified by proteomics and conducted FTSAs with the pyrazolone compounds and the PRAPP. These experiments identified 14-3-3-E as the primary target and 14-3-3-Q as the minor target of the pyrazolone lead compounds. Given the major *T*_m_ shift observed for 14-3-3-E with P_2_ in vitro by FTSA, we conducted CETSA experiments to robustly confirm the cellular drug–target engagement between P_2_ and 14-3-3-E. The CETSA experiment revealed a decrease in the thermal stability of 14-3-3-E in the cell lysate in the presence of P_2_, indicating the potential disruption of protein–protein interactions between 14-3-3-E and its cellular non-14-3-3 binding partners. The functional importance of 14-3-3-E and 14-3-3-Q isoforms in ALS has been extensively studied in the literature using SOD1^G93A^ transgenic mice models and N2a neuroblastoma cells. The 14-3-3-E and 14-3-3-Q isoforms are known to interact with mutant SOD1 aggregates in the JUNQ of N2a neuroblastoma cells that express SOD1^G93A^ and become insoluble, which causes a decrease in cytosolic 14-3-3 abundance, leading to apoptosis. Therefore, we hypothesize that the pyrazolone compounds prevent apoptosis by disrupting 14-3-3-E and 14-3-3-Q interactions with misfolded SOD1^G93A^ aggregates and elevating the cytosolic levels of 14-3-3-E and 14-3-3-Q. This is the first definitive identification of the target(s) of a small molecule with activity in an ALS phenotypic cell-based model and in the ALS mouse model.

## Experimental Section

### Synthesis

#### General Methods

All reactions were carried out under an atmosphere of dry nitrogen in oven-dried glassware unless stated otherwise. Indicated reaction temperatures refer to those of the reaction bath, while room temperature (rt) is noted as about 25 °C. All other solvents were of anhydrous quality and used as received. Commercially available starting materials and reagents were purchased from Aldrich, TCI, and Fisher Scientific and used as received unless specified otherwise. Analytical thin-layer chromatography (TLC) was performed with precoated silica gel 60 F254 plates (5 × 20 cm, 60 Å, 250 μm). Column chromatography was performed with silica gel 60 (230–400 mesh) or C18 reverse phase column with a Combi-flash instrument. ^1^H and ^13^C NMR spectra were recorded in deuterated solvent on a Bruker Avance III 500 MHz spectrometer (direct cryoprobe). Chemical shifts are reported in ppm with the solvent resonance as internal standard (CDCl_3_, 7.27 ppm, 77.23 ppm and DMSO-*d*_6_, 2.5 ppm, 39.51 ppm for ^1^H and ^13^C respectively). Data are reported as follows: chemical shift, multiplicity (s = singlet, d = doublet, dd = doublet of doublet, t = triplet, q = quartet, br = broad, m = multiplet, abq = ab quartet), number of protons, and coupling constants. Low-resolution mass spectra (LRMS) were acquired on an Agilent 1110 MSD. High-resolution mass spectral (HRMS) data were collected in-house using an Agilent 6210 LC-TOF (ESI). All compounds submitted for biological testing were found to be >95% pure by analytical HPLC.

#### 4-Azido-2,3,5,6-tetrafluorobenzaldehyde (**8**)

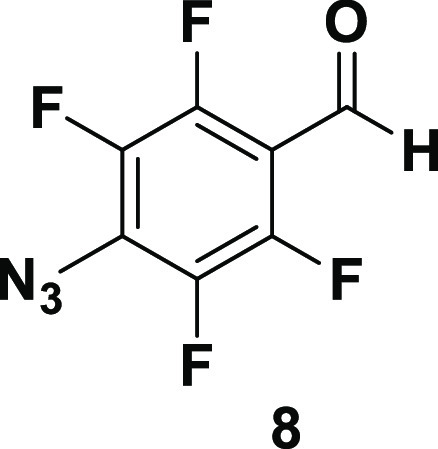



To a stirred solution of 2,3,4,5,6-pentafluorobenzaldehyde (1 g, 5 mmol) in acetone (8 mL) and water (2 mL) was slowly added sodium azide (0.4 g, 6 mmol), and the resulting mixture was refluxed for 12 h. The reaction mixture was cooled, diluted with water (10 mL), and extracted with ether (3 × 15 mL). The combined organic layers were dried over anhydrous sodium sulfate and evaporated to yield a brown oil. The crude product was purified by flash chromatography on silica gel (30% ethyl acetate in hexane) to afford **8** as a brown, viscous oil (0.61 g, 55%). ^1^H NMR (500 MHz, CDCl_3_) δ 10.23 (s, 1H). ^13^C NMR (126 MHz, CDCl_3_) δ: 182.07, 147.49, 141.57, 139.68, 126.87, 111.04. MS-APCI [M – N_2_]^+^ = 190.84.

#### N-(4-Azido-2,3,5,6-tetrafluorobenzyl)prop-2-yn-1-amine (**9**) (IPC2)

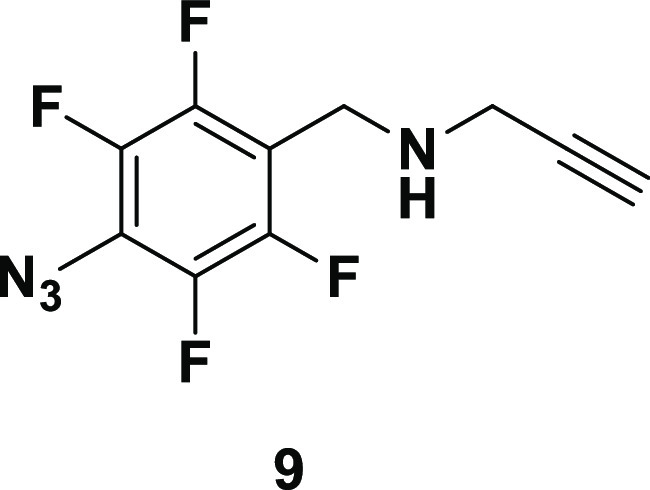



To a stirred solution of **8** (3.42 g, 15.6 mmol) in ethanol (250 mL) were added propargylamine (1.72 g, 31.2 mmol) and glacial acidic acid (0.2 mL), followed by anhydrous sodium sulfate (30 g), and the mixture was stirred for 12 h. The reaction mixture was filtered to remove the anhydrous sodium sulfate, and the resulting imine was cooled to 0 °C using an ice bath. Sodium cyanoborohydride (1.96 g, 31.2 mmol) was added to the resulting solution with stirring and allowed to warm to room temperature. After 2 h, the reaction was quenched with water and concentrated to remove ethanol. The crude product was dissolved in ethyl acetate and washed sequentially with sodium bicarbonate and brine. The organic layer was dried over anhydrous sodium sulfate and concentrated to yield a yellowish-brown viscous oil which was purified by flash chromatography on silica gel (70% ethyl acetate in hexane) to obtain **9** as a yellow solid (1.9 g, 47%). Note: This compound is highly unstable upon exposure to light. The reaction flask and the vials were covered with aluminum foil to avoid exposure to light. ^1^H NMR (500 MHz, CDCl_3_) δ 3.96 (t, *J* = 1.5 Hz, 2H), 3.42 (d, *J* = 2.4 Hz, 2H), 2.22 (t, *J* = 2.4 Hz, 1H) (NH proton was not visible). MS-APCI [M + H]^+^ = 259.17.

#### Ethyl 4-((4-methoxybenzyl)oxy)-3-oxobutanoate (**10**)

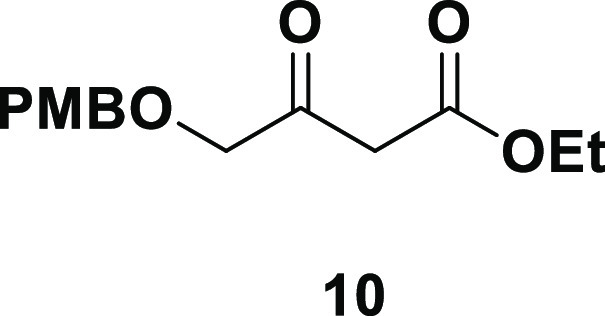



A solution of 4-methoxybenzyl alcohol (16.8 g, 122 mmol) in dry THF (30 mL) was added dropwise over 15 min to a stirred solution of NaH (60% dispersed in mineral oil, 6.1 g, 152 mmol) in THF (70 mL) at 0 °C. After 30 min, a solution of ethyl 4-chloroacetoacetate (10 g, 60 mmol) in dry THF (30 mL) was added dropwise over 15 min at 0 °C, and the mixture was allowed to warm to rt with stirring for 12 h. The reaction was quenched with cold 1 N HCl (25 mL) and diluted with deionized H_2_O (100 mL). The aqueous layer was extracted with ethyl acetate (3 × 150 mL). The combined organic extracts were washed with brine (1 × 200 mL), filtered through Celite, and concentrated to give a red oil. Purification by flash chromatography on silica gel yielded the desired product as a colorless oil (10.2 g, 63%). ^1^H NMR (500 MHz, CDCl_3_) δ 7.28–7.20 (m, 2H), 6.90–6.84 (m, 2H), 4.50 (s, 2H), 4.16 (q, *J* = 7.1 Hz, 2H), 4.09 (s, 1H), 3.80 (s, 3H), 3.50 (s, 2H), 1.23 (t, *J* = 7.1 Hz, 3H). ^13^C NMR (126 MHz, CDCl_3_) δ: 201.88, 167.02, 159.58, 129.60, 129.47, 128.97, 113.95, 113.90, 74.52, 73.17, 61.41, 55.30, 46.08, 14.07. MS-APCI [M + H]^+^ = 267.14.

#### 5-(((4-Methoxybenzyl)oxy)methyl)-1,2-dihydro-3H-pyrazol-3-one (**11**)

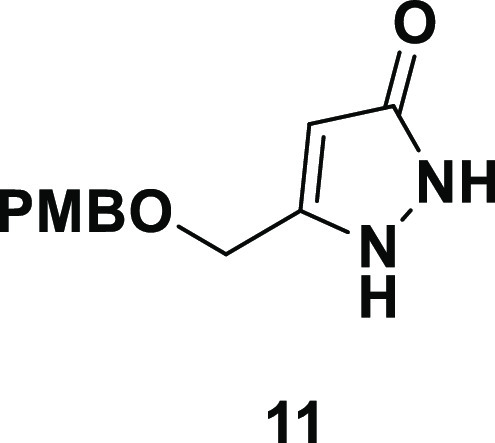



To a stirred suspension of **10** (10.5 g, 94 mmol) in ethanol (50 mL) was added hydrazine (1.9 g, 59 mmol) dropwise, and a white precipitate formed within 5–10 min. The resulting precipitate was filtered, washed with cold ethanol, and dried under high vacuum to afford **11** as a white powder (8.2 g, 89%). ^1^H NMR (500 MHz, DMSO) δ: 7.25 (d, *J* = 8.5 Hz, 2H), 6.90 (d, *J* = 8.7 Hz, 2H), 4.39 (s, 2H), 4.32 (s, 2H), 3.74 (s, 4H). (NH protons are not visible). ^13^C NMR (126 MHz, DMSO) δ: 159.20, 130.50, 129.77, 114.10, 71.29, 55.52. MS-APCI [M + H]^+^ = 235.12.

#### 1,1′-(5-(((4-Methoxybenzyl)oxy)methyl)-3-oxo-1H-pyrazole-1,2(3H)-diyl)bis(ethan-1-one) (**12**)

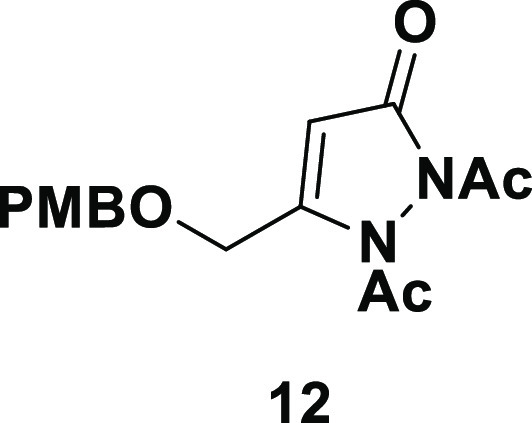



Acetyl chloride (3.7 g, 47 mmol) in dry dichloromethane (50 mL) was cooled to −40 °C, and 2,6-leutidine (5.7 g, 53 mmol) was added to it all at once (deep amber color appeared). Immediately after the addition of **11**, the reaction was stirred for 5 h at −40 °C, and the stirring was continued overnight at room temperature. The solvent was evaporated, and the crude product was dissolved in ethyl acetate (50 mL) and washed sequentially with 10% citric acid (2 × 15 mL) and brine (15 mL). The organic layer was dried over anhydrous sodium sulfate and evaporated to yield a yellow oil. The crude product was purified by flash chromatography on silica gel (25% ethyl acetate in hexane) to yield **12** as a colorless oil (4.5 g, 66%). ^1^H NMR (500 MHz, DMSO) δ 7.28 (d, *J* = 8.6 Hz, 2H), 6.91 (d, *J* = 8.6 Hz, 2H), 6.37 (s, 1H), 4.47 (s, 2H), 4.43 (s, 2H), 3.75 (s, 3H), 2.56 (s, 3H), 2.30 (s, 3H). MS-APCI [M + H]^+^ = 319.42.

#### 1,1′-(5-(Hydroxymethyl)-3-oxo-1H-pyrazole-1,2(3H)-diyl)bis(ethan-1-one) (**13**)

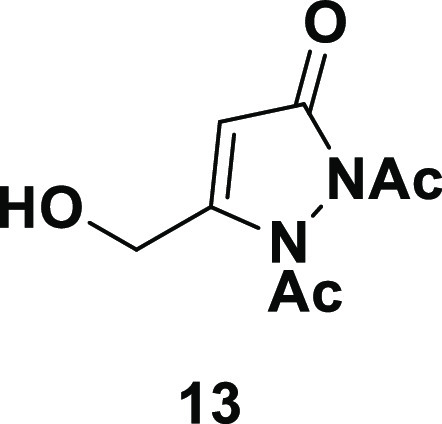



To a stirred solution of **12** (500 mg, 1.57 mmol) and anisole (510 mg, 4.71 mmol) in dry dichloromethane (5 mL) cooled in an ice bath was added TFA (269 mg, 2.36 mmol), followed by triflic acid (118 mg, 0.78 mmol) dropwise, and the reaction was allowed to proceed for 15 min. The solvent was evaporated (without heating), and the crude product was dissolved in dichloromethane (2 mL) and purified by flash chromatography on silica gel (80% ethyl acetate in hexane) to afford **13** as a colorless oil (280 mg, 90%). ^1^H NMR (500 MHz, CDCl_3_) δ 6.40 (s, 1H), 4.76 (d, *J* = 0.8 Hz, 2H), 2.67 (s, 3H), 2.33 (s, 3H). ^13^C NMR (126 MHz, CDCl_3_) δ: 171.85, 167.37, 156.42, 147.47, 103.20, 22.86. MS-APCI [M + H]^+^ = 198.92.

#### 1,1′-(5-(Bromomethyl)-3-oxo-1H-pyrazole-1,2(3H)-diyl)bis(ethan-1-one) (**14**)

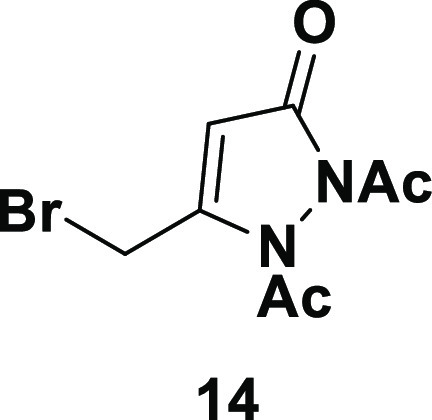



To a stirred solution of **13** (310 mg, 1.5 mmol) in dry dichloromethane (8 mL) was added triphenylphosphine (739 mg, 2.82 mmol), followed by carbon tetrabromide (934 mg, 2.82 mmol), and stirring continued for 12 h. The reaction mixture was concentrated, directly injected onto the silica gel column, and purified by flash chromatography (20% ethyl acetate in hexane) to yield **14** as a colorless oil (256 mg, 63%). ^1^H NMR (500 MHz, CDCl_3_) δ: 6.53 (d, *J* = 0.8 Hz, 1H), 4.78 (d, *J* = 0.8 Hz, 2H), 2.65 (s, 3H), 2.33 (s, 3H). ^13^C NMR (126 MHz, CDCl_3_) δ: 171.11, 167.77, 156.19, 144.18, 105.45, 23.45, 22.79, 21.27. MS-APCI [M + H]^+^ = 162.02.

#### 1,1′-(5-(((4-Azido-2,3,5,6-tetrafluorobenzyl)(prop-2-yn-1-yl)amino)methyl)-3-oxo-1H-pyrazole-1,2(3H)-diyl)bis(ethan-1-one) (**15**) (IPC1)

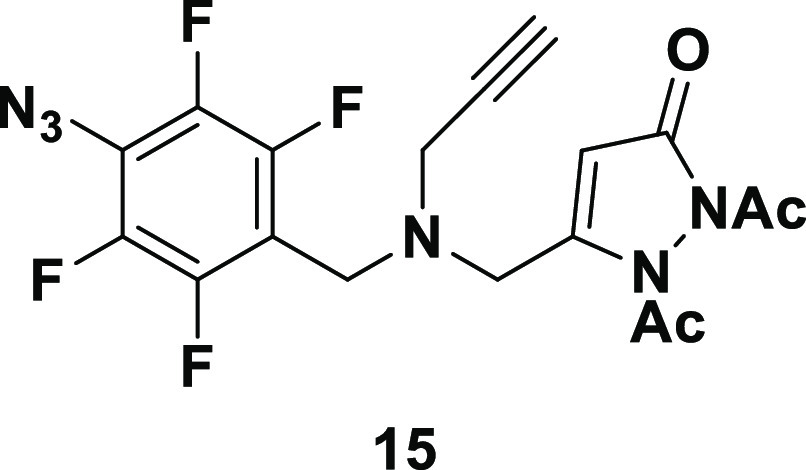



To a stirred solution of **9** (355 mg, 1.3 mmol) and **14** (212 mg, 0.8 mmol) in dry DMF (0.3 mL) was added potassium carbonate (224 mg, 1.6 mmol), and the resulting mixture was stirred for 12 h at r.t. The reaction mixture was diluted with ethyl acetate (10 mL) and washed with brine (3 × 2 mL). The organic layer was dried over anhydrous sodium sulfate and evaporated to yield a yellow oil. The crude product was purified by flash chromatography on silica gel (2% ethyl acetate in dichloromethane) to obtain compound **15** as a light yellow solid (120 mg, 33%). Note: The reaction flask and vials were covered with aluminum foil to avoid exposure to light. ^1^H NMR (500 MHz, CDCl_3_) δ 6.42 (d, *J* = 1.1 Hz, 1H), 4.12–4.10 (m, 2H), 3.88 (t, *J* = 1.5 Hz, 2H), 3.43 (d, *J* = 2.4 Hz, 2H), 2.61 (s, 3H), 2.32 (s, 3H), 2.28 (t, *J* = 2.3 Hz, 1H). ^13^C NMR (126 MHz, CDCl_3_) δ: 171.09, 167.95, 156.63, 146.50, 145.29, 141.85, 119.90, 112.43, 112.29, 112.15, 103.80, 74.44, 50.58, 45.46, 43.09, 23.60, 21.29. MS-APCI [M + H]^+^ = 440.24.

#### 5-(((4-Azido-2,3,5,6-tetrafluorobenzyl)(prop-2-yn-1-yl)amino)methyl)-1,2-dihydro-3H-pyrazol-3-one (**16**) (PRAPP)

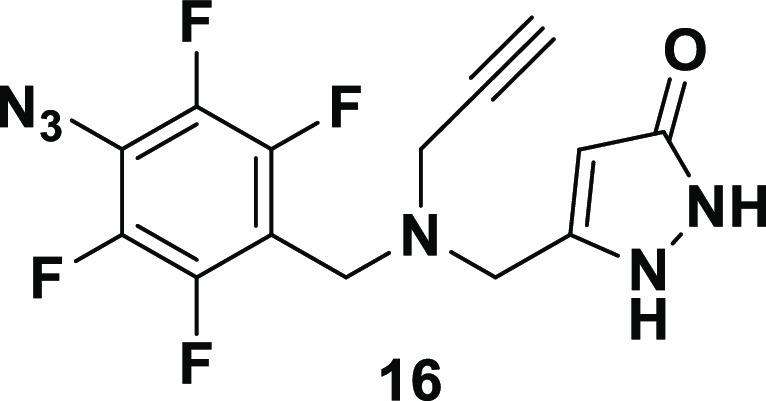



To a stirred solution of **15** (92 mg, 0.21 mmol) in ethanol (0.6 mL) was added TFA (0.36 g, 3.1 mmol) dropwise and stirred for 5 h at 65 °C. The reaction mixture was concentrated, and the product was purified by flash chromatography on C18 silica gel (reverse phase, 100% acetonitrile) to afford **16** as a white solid (36 mg, 48%). ^1^H NMR (500 MHz, DMSO) δ: 11.42 (s, 1H), 9.36 (s, 1H), 5.35 (s, 1H), 3.71 (s, 2H), 3.55 (s, 2H), 3.28 (d, *J* = 2.3 Hz, 2H), 3.23 (d, *J* = 2.7 Hz, 1H). ^13^C NMR (126 MHz, DMSO) δ: 166.74, 161.27, 146.16, 144.29, 143.92, 140.90, 118.98, 111.93, 89.79, 78.04, 76.50, 51.13, 44.00, 41.56. MS-APCI [M + H]^+^ = 355.48. Analytical HPLC UV–vis: purity_254 nm_ = 98%.

### Neuroprotection against MG-132-Induced Toxicity in PC12 Cells Expressing Mutant SOD1^G93A^

Viability EC_50_ values were determined according to the previously reported assay procedure.^43^ Briefly, PC12 cells were seeded at 15,000 cells/well in 96-well plates, and the medium was changed to remove the doxycycline 4 days prior to compound addition. Compounds were assayed in 12-point dose–response experiments to determine the potency and efficacy. The highest compound concentration tested was 32 μM, which was decreased by half with each subsequent dose. After a 24 h incubation with the analytes, MG-132 (400 nM) was added and the cells were incubated at 37 °C for 24 h. The cells were washed twice with PBS. Calcein-AM was added at a final concentration of 1 μM for 20 min at room temperature, and the fluorescence intensity was read in a POLARstar fluorescence plate reader (BMG). EC_50_ values were determined by standardizing fluorescent signal to DMSO treated controls. Interplate variability was corrected via standardization using a well-characterized positive control (P_2_, EC_50_ = 67 nM). The presented data represent the mean average of duplicate experiments.

### Characterization of PRAPP Photolysis

The PRAPP (100 μM) was dissolved in PBS (50 mM, pH 7.4 [1% DMSO v/v]) and irradiated with UV light (λ = 312 nm, 120 V, 60 Hz, 0.2 A [Spectroline model EB-160C], distance ∼4 cm). UV light was removed from the reaction mixtures at varying time points (1, 2, 5, 10, and 20 min) and immediately analyzed by HPLC-UV–vis_278 nm_ (injection volume of 5 μL). PRAPP % remaining was determined based on the area under the curve (AUC) of the UV–vis_278 nm_ chromatogram. A first-order exponential decay curve was generated based on the AUC of the UV–vis chromatogram at 0, 1, 2, 5, 10, and 20 min and used to determine *t*_1/2_ of photolysis.

### General Procedure for Photoaffinity Labeling

PC12-SOD1^G93A^ cells were cultured and grown to 90% confluency (in 6-well plates) and washed once with PBS followed by the addition of serum medium containing the appropriate analyte at the indicated concentrations. Three incubation scenarios were studied (at 37 °C): Scenario 1) 12 h preincubation with PRAPP in the absence of MG-132; Scenario 2) 12 h preincubation with PRAPP in the absence of MG-132 followed by an additional 24 h incubation (control); and Scenario 3) 12 h preincubation with PRAPP followed by the addition of MG-132 (100 nM) and an additional 24 h incubation. After incubation, the medium was discarded and cells were washed with PBS (1 × 300 μL). Fresh serum-free medium was added, and the cells were irradiated with UV light (312 nm, 120 V, 60 Hz, 0.2 A [Spectroline model EB-160C], distance ∼4 cm) for 15 min. The medium was removed, cells were washed with PBS (2 × 500 μL), and 200 μL of lysis buffer containing Tris HCl (25 mM, pH 7.6), NaCl (150 mM), 1% NP40, 1% sodium deoxycholate, 0.1% SDS, and protease inhibitor cocktail (Sigma-Aldrich, 1:100 v/v) was added to each well. Complete lysis was observed by a microscope after 10 min at 4 °C with agitation. Each lysate was centrifuged at 4 °C for 5 min at 13,000 rpm, and the resulting supernatant was concentrated with a centrifugal filter (Amicon Ultra Centrifugal Filters 3K cutoff, Millipore), normalized to 1.0 mg/mL by the Bradford assay, and analyzed immediately or stored at −80 °C for subsequent analysis. Treatment Scenario 1 was used for competition experiments with 30 μM PRAPP and varying concentrations of the competitor (P_1_, P_2_, P_3_, and edaravone) as shown in Figure 5, Figure S4, Figure S5, and Figure S6.

### General Procedure for Visualization Using N_3_–Alexa-647

In a darkened (wrapped with aluminum foil) Eppendorf tube, each lysate (30 μL [30 μg]) was incubated with 10 μL of the freshly prepared “click” premix containing CuSO_4_ (5 mM), sodium ascorbate (5 mM), TBTA (200 μM), and N_3_–Alexa-647 (50 μM) were added to PBS (50 mM, pH 7.4). The corresponding final concentrations within the lysate click incubation were: CuSO_4_ (1.25 mM), sodium ascorbate (1.25 mM), TBTA (50 μM), and N_3_–Alexa-647 (12.5 μM). The mixture was vortexed briefly and incubated for 1.5 h at 37 °C in the dark. NuPAGE LDS sample loading buffer (4 × 13 μL) was added, and the mixture was denatured in boiling water for 10 min. SDS-PAGE was performed on Novex Bis-Tris gel 4–12% with MES SDS running buffer (90 V constant for 30 min followed by 100 V for 90 min; load volume = 25 μL). Protein standards that gave appropriate visible and fluorescent bands for our application included the BioRad Precision Plus All Blue Protein Standard and Novex Pre-Stained Protein Standard. In-gel fluorescence was monitored (excitation λ = 635 nm; emission λ = 670 nm) using a Typhoon 9400 (GE Healthcare) fluorimager. Densitometric analysis and image refinement were carried out as needed using ImageJ software (NIH, open source). The contrast and brightness of images were modified using ImageJ and/or Microsoft Word to enhance visualization for publication purposes.

### General Procedure for Protein Pull-down Using Dde TAMRA Biotin Azide

Background biotin was removed by nutrating the appropriate lysate (200 μL [1 mg, 5 mg/mL]) with streptavidin bound magnetic beads [Dynabeads MyOne Streptavidin T1, Life Technologies] (50 μL) for 1 h. A magnet was used to separate the Dynabeads from the supernatant. The supernatant was collected, and the beads were washed with PBS (1 × 50 μL). The combined supernatant/PBS wash fractions (approximately 250 μL total volume) were incubated with 250 μL of the freshly prepared “click” premix containing the following components: CuSO_4_ (5 mM), sodium ascorbate (5 mM), TBTA (800 μM), and Dde-TAMRA biotin azide conjugate (600 μM [Click chemistry tools]) in PBS (50 mM, pH 7.4). Correspondingly, final concentrations within the lysate click incubation were as follows: CuSO_4_ (2.5 mM), sodium ascorbate (2.5 mM), TBTA (400 μM), and TAMRA desthiobiotin azide (300 μM). The mixture was vortexed briefly and incubated for 3 h at 37 °C (for the control experiment, 250 μL of PBS buffer was used). The excess Dde-TAMRA biotin azide was removed using a centrifugal filter (Amicon Ultra Centrifugal Filter 10 K cutoff × 3 [alternatively, acetone precipitation was also used]). The resulting solution was nutrated with Dynabeads (200 μL) for 1 h. The beads were separated using a magnet, washed with PBS (2 × 400 μL), 0.1% Triton X-100 in PBS (2 × 400 μL), 1% SDS in PBS (400 μL), and PBS (400 μL) before being suspended in PBS/LDS loading buffer (50 μL:50 μL), and the beads were cleaved in boiling water for 10 min (the hydrazine cleavage protocol did not produce good results).^44^ The beads were separated, and the supernatant was collected. The supernatant was loaded onto a Novex Bis-Tris gel 4–12% and run with MES SDS running buffer (90 V constant for 30 min followed by 100 V for 90 min) (load volume = 25 μL). The excision of the 25 kDa protein band was guided by TAMRA fluorescence and subjected to proteomics analysis. A separate pull-down experiment was also performed using biotin-PEG3-azide (instead of TAMRA-Dde).

### Proteomic Analysis

The excised protein bands of interest were digested with sequencing-grade trypsin. The samples were loaded directly onto a PepMap C18 cartridge (0.3 mm × 5 mm, 5 μm particle) trap column and then a 75 μm × 150 mm PepMap C18 analytical column (Thermo Fisher Scientific) and separated at a flow rate of 300 nL/min. Solvent A was 0.1% formic acid in water, and solvent B was 0.1% formic acid and 80% acetonitrile in water. The 75 min solvent gradient of LC was 5% B from 0 to 3 min, 5–35% B to 55 min, 35–95% B to 60 min, and washed 95% to 65 min, followed by 5% B equilibration to 75 min. The peptides were directly eluted into a Q Exactive HF mass spectrometer, and the full MS scans were acquired in the Q-Exactive mass spectrometer over a 350–1400 *m*/*z* range with a resolution of 120,000 (at 400 *m*/*z*) from 5 to 63 min. The tandem mass spectrum was acquired in the mass analyzer with a resolution of 30,000 (at 400 *m*/*z*).

All MS/MS samples were analyzed using Mascot (Matrix Science, London, UK; version 2.6.2). Mascot was set up to search the Uniprot database (downloaded on 15/10/2019, containing 29,942 entries), assuming the digestion enzyme trypsin. Mascot was searched with a fragment ion mass tolerance of 0.30 Da, and a parent ion tolerance of 20 ppm of carbamidomethyl of cysteine was specified in Mascot as a fixed modification. Deamidation of asparagine and glutamine and oxidation of methionine were specified in Mascot as variable modifications. Scaffold (version Scaffold_4.11.1, Proteome Software Inc., Portland, OR) was used to validate MS/MS-based peptide and protein identifications. Peptide identifications were accepted if they could be established at greater than 96.0% probability by the Peptide Prophet algorithm^45,46^ with Scaffold delta-mass correction. Protein identifications were accepted if they could be established at a greater than 32.0% probability of achieving a false discovery rate (FDR) less than 5.0% and contained at least two identified peptides. Protein probabilities were assigned by the Protein Prophet algorithm. Proteins that contained similar peptides and could not be differentiated based on MS/MS analysis alone were grouped to satisfy the principles of parsimony. Proteins sharing significant peptide evidence were grouped into clusters.

### Protein Expression and Purification

His-tagged 14-3-3-E, B, and H plasmids were purchased from Addgene (plasmid ID# 31562, 39128, and 38814, respectively). *E. coli* (BL21) were transformed with extracted plasmids via heat shock and plated on selected media. Individual colonies were selected, grown, and sequenced to confirm gene presence. 1 L cultures were grown to an OD600 of 0.6 at 37 °C and 220 rpm. Protein expression was induced with 1 mM IPTG and expressed for 12 h at 100 rpm and room temperature (approximately 25 °C). Cells were spun down at 4000 rpm for 15 min at 4 °C and resuspended in lysis buffer containing 50 mM Tris (7.4), 150 mM NaCl, 0.08% DDM, 5 mM imidazole, and 5% glycerol (with an EDTA-free complete mini protease inhibitor cocktail). Cells were lysed via sonication, and the lysate was separated from cell debris by centrifugation at 15,000 rpm, 4 °C, for 45 min. The His-tagged protein was purified using QIAGEN Ni-NTA superflow columns, and the His-tag was removed with TEV protease. His-tag-removed proteins were further purified by size exclusion chromatography. His tag removal and size exclusion chromatography purification were done at the Northwestern University Protein Production core. 14-3-3-Q and 14-3-3-G proteins were purchased from Abchem (catalog numbers ab85270 and ab53869, respectively).

### Fluorescent Thermal Shift Assay (FTSA) Protocol

All of the FTSA experiments were conducted in HEPES buffer (60 mM HEPES, 150 mM NaCl, pH 7.4). A DMSO balanced 8-point dilution series of the compound in HEPES buffer was prepared in a 96-well assay plate (200, 100, 50, 25, 12.5, 6.25, 3.125, and 0 μM). Then, a 5 μL volume of the dilution series was transferred to a 384-well PCR plate using a multichannel pipet, and the plate was centrifuged at 1000 rpm for 2 min (the plate was covered with an adhesive aluminum seal). The protein sample was premixed at a concentration of 0.04 μg/μL with a 5X concentration of SYPRO orange in HEPES buffer, and a 5 μL aliquot was added to the compound wells. The plate was covered with an optical adhesive seal and was shaken for 2 min, followed by centrifugation at 1000 rpm for 2 min. The thermal scan was performed from 20 to 90 °C with a temperature gradient of 0.5 °C/min. The fluorescence was measured on a Bio-Rad CFX384 real-time PCR instrument.

### Cellular Thermal Shift Assay (CETSA) Protocol

Before the experiments were initiated, PC12-SOD1^G93A^ YFP cells were maintained in DMEM high-glucose media supplemented with 10% horse serum, 5% Tet-approved FBS, 1% Pen-strep, 1X l-glutamine, 200 μg/mL hygromycin, and 100 μg/mL G418. On the day preceding the experiments, the cells were plated onto two separate 10 cm dishes at a concentration of 10 × 10^6^ cells per 10 mL of the supplemented media. They were then incubated overnight at 37 °C in a CO_2_ incubator (5% CO_2_) until they achieved 90–95% confluency.

The cell medium was subsequently removed, and the cells were rinsed with 1X PBS before being detached using 1 mL of 1X trypsin. To this, 5 mL of 1X PBS solution was added, and the cells were then resuspended, transferred to two separate 15 mL conical tubes, and centrifuged at 1100 rpm for 3 min. The supernatant was discarded, and the cells were resuspended in 1 mL of 1X PBS containing a proteasomal inhibitor cocktail (EDTA-free) (Sigma-Aldrich, 1:100 v/v). This solution was then transferred to two Eppendorf tubes and immediately snap-frozen by using liquid nitrogen. The cells underwent lysis through four freeze/thaw cycles, alternating between liquid nitrogen and heating at 30 °C. The cell debris was then separated from the supernatant by centrifuging at 19,000*g* for 20 min at 4 °C. The supernatants from both tubes were combined and split between two new Eppendorf tubes (950 μL per tube). The cell lysates were subsequently treated with either P_2_ (500 μM) or DMSO (1% v/v) and incubated for 2 h at room temperature. Each sample was further divided into seven smaller aliquots (120 μL each), heated for 3 min at specified temperatures, and then allowed to cool at room temperature for another 3 min. After that, the lysate samples were centrifuged at 19,000*g* for 20 min at 4 °C, and the soluble fraction was collected. Subsequently, 6 μL of 5X SDS loading buffer was added to 24 μL of each sample, and the resulting mixture was denatured in boiling water for 10 min. Finally, 20 μL of this mixture was loaded onto an SDS PAGE gel.

SDS PAGE was conducted using a 15-well Novex Bis-Tris 4–12% prepacked gel and 1X MES-SDS gel running buffer (comprising 50 mM MES, 50 mM Tris, 1 mM EDTA, and 0.1% SDS) at a constant voltage of 100 mV over a duration of 2 h. Postelectrophoresis, the gel was transferred to a nitrocellulose membrane at 4 °C using a constant current of 400 mA in Tris-glycine transfer buffer (made from 800 mL H_2_O, 200 mL MeOH, 25 mM Tris, and 190 mM glycine). The nitrocellulose membrane was then blocked with NFD-milk solution (1X PBS with 2.5% nonfat dry milk) and left overnight at 4 °C. After that, the membrane was rinsed with 1X PBS/0.1% Tween 20 solution (3 × 5 min), at room temperature, and incubated for 1 h at room temperature with the 14-3-3-E primary antibody (α-rabbit, Invitrogen, 1:1000 dilution in 1X PBS with 2.5 mg/mL BSA). The primary antibody solution was removed, and the membrane was washed with 1X PBS/0.1% Tween 20 (3 × 5 min). The membrane was then incubated with the secondary antibody solution (α-rabbit, horseradish peroxidase-linked, 1:5000 dilution in NFD-milk blocking solution) for 1 h. After that, the secondary antibody solution was removed, and the membrane was washed again with 1X PBS/0.1% Tween 20 (3 × 5 min). The membrane was then incubated with a solution from the PerkinElmer chemiluminescence enhancement kit for 5 min at room temperature. Immunoblot raw image data were captured using an Azure Sapphire Biomolecular Imager with a 4-second exposure. These raw immunoblot images were analyzed by using ImageJ software. Data (expressed as relative band intensity %) were normalized relative to the samples that were incubated at the lowest temperature. The ITDR CETSA experiment was conducted similarly to the aforementioned protocol at 63 °C, and the data were normalized relative to the DMSO control where [P_2_] = 0

## Supporting Information Available

Visualization protocol for covalently modified proteins, effect of MG-132 on 25 kDa covalently modified band intensity, supplemental figures related to competition studies of PRAPP, fluoresence-guided gel excision, supplemental figures related to FTSA studies of PRAPP, P2 and P1 with 14-3-3 isoforms, ^1^H NMR spectra and proteomics analysis results table (PDF)

## References

[ref1] Brown R. W. J. The Motor Neuron Diseases. In Harrison’s Principles of Internal Medicine; Fauchi A. S.,, Eds.; McGraw-Hill: New York, 1998, p 2368–71.

[ref2] Rowland L. P.; Shneider N. A. Amyotrophic lateral sclerosis. N Engl J. Med. 2001, 344, 1688–700. 10.1056/NEJM200105313442207. 11386269

[ref3] Abhinav K.; Stanton B.; Johnston C.; Hardstaff J.; Orrell R. W.; Howard R.; et al. Amyotrophic lateral sclerosis in South-East England: A population-based study. The South-East England register for amyotrophic lateral sclerosis (SEALS Registry). Neuroepidemiology 2007, 29, 44–8. 10.1159/000108917. 17898523

[ref4] Greenway M. J.; Andersen P. M.; Russ C.; Ennis S.; Cashman S.; Donaghy C.; et al. A.N.G. mutations segregate with familial and ’sporadic’ amyotrophic lateral sclerosis. Nat. Genet. 2006, 38, 411–3. 10.1038/ng1742. 16501576

[ref5] Ticozzi N.; Vance C.; Leclerc A. L.; Keagle P.; Glass J. D.; McKenna-Yasek D.; et al. Mutational analysis reveals the F.U.S. homolog TAF15 as a candidate gene for familial amyotrophic lateral sclerosis. Am. J. Med. Genet B Neuropsychiatr Genet 2011, 156B, 285–90. 10.1002/ajmg.b.31158. 21438137

[ref6] Rosen D. R.; et al. Mutations in Cu/Zn superoxide dismutase gene are associated with familial amyotrophic lateral sclerosis. Nature 1993, 362, 59–62. 10.1038/362059a0. 8446170

[ref7] Vance C.; et al. Mutations in F.U.S., an R.N.A. processing protein, cause familial amyotrophic lateral sclerosis type 6. Science 2009, 323, 1208–1211. 10.1126/science.1165942. 19251628 PMC4516382

[ref8] Sreedharan J.; Blair I. P.; Tripathi V. B.; Hu X.; Vance C.; Rogelj B.; Ackerley S.; Durnall J. C.; Williams K. L.; Buratti E.; Baralle F.; de Belleroche J.; Mitchell J. D.; Leigh P. N.; Al-Chalabi A.; Miller C. C.; Nicholson G.; Shaw C. E. TDP-43 mutations in familial and sporadic amyotrophic lateral sclerosis. Science 2008, 319, 1668 10.1126/science.1154584. 18309045 PMC7116650

[ref9] Maruyama H.; et al. Mutations of optineurin in amyotrophic lateral sclerosis. Nature 2010, 465, 223–226. 10.1038/nature08971. 20428114

[ref10] Deng H. X.; et al. Mutations in UBQLN2 cause dominant X-linked juvenile and adult-onset ALS. and ALS./dementia. Nature 2011, 477, 211–215. 10.1038/nature10353. 21857683 PMC3169705

[ref11] Fecto F.; Deng H.-X.; Siddique T. Discovering the Connection between Familial and Sporadic Amyotrophic Lateral Sclerosis: Pathology Trumps Genetics. Future Neurol. 2010, 5 (5), 625–628. 10.2217/fnl.10.47.

[ref12] Cleveland D. W.; Laing N.; Hurse P. V.; Brown R. H. Jr. Toxic mutants in Charcot’s sclerosis. Nature 1995, 378, 342–343. 10.1038/378342a0. 7477368

[ref13] Gill C.; Phelan J. P.; Hatzipetros T.; Kidd J. D.; Tassinari V. R.; Levine B.; Wang M. Z.; Moreno A.; Thompson K.; Maier M.; Grimm J.; Gill A.; Vieira F. G. SOD1-Positive Aggregate Accumulation in the CNS Predicts Slower Disease Progression and Increased Longevity in a Mutant SOD1Mouse Model of ALS. Sci. Rep. 2019, 9 (1), 6724 10.1038/s41598-019-43164-z. 31040321 PMC6491559

[ref14] Matsumoto G.; Stojanovic A.; Holmberg C.; Kim S.; Morimoto R. I. Structural Properties and Neuronal Toxicity of ALS-Associated SOD1. Aggregates. J. Cell Biol. 2005, 171, 75–85. 10.1083/jcb.200504050. 16216923 PMC2171239

[ref15] Matsumoto G.; Kim S.; Morimoto R. I. Huntingtin and Mutant SOD1 form Aggregate Structures with Distinct Molecular Properties in Human Cells. J. Biol. Chem. 2006, 281, 4477–4485. 10.1074/jbc.M509201200. 16371362

[ref16] Gidalevitz T.; Krupinski T.; Garcia S. M.; Morimoto R. I. Toxicity of Mutant SOD1 is Directed by Protein Polymorphisms. PLoS Genetics 2009, 5 (3), e1000399 10.1371/journal.pgen.1000399. 19266020 PMC2642731

[ref17] Lobsiger C. S.; Cleveland D. W. Glial Cells as Intrinsic Components of Non-Cell-Autonomous Neurodegenerative Disease. Nat. Neurosci. 2007, 10 (11), 1355–1360. 10.1038/nn1988. 17965655 PMC3110080

[ref18] Harraz M. M.; Marden J. J.; Zhou W.; Zhang Y.; Williams A.; Sharov V. S.; Nelson K.; Luo M.; Paulson H.; Schoneich C.; Engelhardt J. F. SOD1 mutations disrupt redox-sensitive Rac regulation of NADPH oxidase in a familial ALS. model. J. Clin. Invest. 2008, 118, 659–670. 10.1172/JCI34060. 18219391 PMC2213375

[ref19] Nishitoh H.; Kadowaki H.; Nagai A.; Maruyama T.; Yokota T.; Fukutomi H.; Noguchi T.; Matsuzawa A.; Takeda K.; Ichijo H. ALS-Linked Mutant SOD1 Induces ER Stress- and ASK1-Dependent Motor Neuron Death by Targeting Derlin-1. Genes Dev. 2008, 22 (11), 1451–1464. 10.1101/gad.1640108. 18519638 PMC2418582

[ref20] Bruijn L. I.; et al. ALS-linked SOD1 mutant G85R mediates damage to astrocytes and promotes rapidly progressive disease with SOD1-containing inclusions. Neuron 1997, 18, 327–338. 10.1016/S0896-6273(00)80272-X. 9052802

[ref21] Howland D. S.; et al. Focal loss of the glutamate transporter EAAT2 in a transgenic rat model of SOD1 mutant-mediated amyotrophic lateral sclerosis (ALS). Proc. Natl. Acad. Sci. USA 2002, 99, 1604–1609. 10.1073/pnas.032539299. 11818550 PMC122237

[ref22] Lee Y.; et al. Oligodendroglia metabolically support axons and contribute to neurodegeneration. Nature 2012, 487, 443–448. 10.1038/nature11314. 22801498 PMC3408792

[ref23] Xu X.; Shen D.; Gao Y.; Zhou Q.; Ni Y.; Meng H.; Shi H.; Le W.; Chen S.; Chen S. A Perspective on Therapies for Amyotrophic Lateral Sclerosis: Can Disease Progression Be Curbed?. Transl. Neurodegener. 2021, 10 (1), 29 10.1186/s40035-021-00250-5. 34372914 PMC8353789

[ref24] Trippier P. C.; Zhao K. T.; Fox S. G.; Schiefer I. T.; Benmohamed R.; Moran J.; Kirsch D. R.; Morimoto R. I.; Silverman R. B. Proteasome Activation Is a Mechanism for Pyrazolone Small Molecules Displaying Therapeutic Potential in Amyotrophic Lateral Sclerosis. ACS Chem. Neurosci. 2014, 5 (9), 823–829. 10.1021/cn500147v. 25001311 PMC4176317

[ref25] Smith E.; Collins I. Photoaffinity Labeling in Target- and Binding-Site Identification. Future Med. Chem. 2015, 7 (2), 159–183. 10.4155/fmc.14.152. 25686004 PMC4413435

[ref26] Seneviratne U.; Huang Z.; am Ende C. W.; Butler T. W.; Cleary L.; Dresselhaus E.; Evrard E.; Fisher E. L.; Green M. E.; Helal C. J.; Humphrey J. M.; Lanyon L. F.; Marconi M.; Mukherjee P.; Sciabola S.; Steppan C. M.; Sylvain E. K.; Tuttle J. B.; Verhoest P. R.; Wager T. T.; Xie L.; Ramaswamy G.; Johnson D. S.; Pettersson M. Photoaffinity Labeling and Quantitative Chemical Proteomics Identify LXRβ as the Functional Target of Enhancers of Astrocytic ApoE. Cell Chem. Biol. 2021, 28 (2), 148–157.e7. 10.1016/j.chembiol.2020.09.002. 32997975

[ref27] Pozdnyakov N.; Murrey H. E.; Crump C. J.; Pettersson M.; Ballard T. E.; am Ende C. W.; Ahn K.; Li Y.-M.; Bales K. R.; Johnson D. S. γ-Secretase Modulator (GSM) Photoaffinity Probes Reveal Distinct Allosteric Binding Sites on Presenilin. J. Biol. Chem. 2013, 288 (14), 9710–9720. 10.1074/jbc.M112.398602. 23396974 PMC3617273

[ref28] Bell J. L.; Haak A. J.; Wade S. M.; Sun Y.; Neubig R. R.; Larsen S. D. Design and Synthesis of Tag-Free Photoprobes for the Identification of the Molecular Target for CCG-1423, a Novel Inhibitor of the Rho/MKL1/SRF Signaling Pathway. Beilstein J. Org. Chem. 2013, 9, 966–973. 10.3762/bjoc.9.111. 23766813 PMC3678708

[ref29] Chen X.; Wang Y.; Ma N.; Tian J.; Shao Y.; Zhu B.; Wong Y. K.; Liang Z.; Zou C.; Wang J. Target identification of natural medicine with chemical proteomics approach: probe synthesis, target fishing and protein identification. Signal Transduct Target Ther 2020, 5 (1), 72 10.1038/s41392-020-0186-y. 32435053 PMC7239890

[ref30] Eirich J.; Orth R.; Sieber S. A. Unraveling the protein targets of vancomycin in living S. aureus and E. faecalis cells. J. Am. Chem. Soc. 2011, 133 (31), 12144–53. 10.1021/ja2039979. 21736328

[ref31] Wang D. Y.; Cao Y.; Zheng L. Y.; Lv D. Y.; Chen L. D.; Xing X. R.; Zhu Z. Y.; Li X. Y.; Chai Y. F. Identification of Annexin A2 as a target protein for plant alkaloid matrine. Chem. Commun. 2017, 53 (36), 5020–5023. 10.1039/C7CC02227A. 28428997

[ref32] Yang X.; Lee W. H.; Sobott F.; Papagrigoriou E.; Robinson C. V.; Grossmann J. G.; Sundström M.; Doyle D. A.; Elkins J. M. Structural Basis for Protein-Protein Interactions in the 14–3-3 Protein Family. Proc. Natl. Acad. Sci. U. S. A. 2006, 103 (46), 17237–17242. 10.1073/pnas.0605779103. 17085597 PMC1859916

[ref33] Sluchanko N. N.; Gusev N. B. Oligomeric Structure of 14–3-3 Protein: What Do We Know about Monomers?. FEBS Lett. 2012, 586 (24), 4249–4256. 10.1016/j.febslet.2012.10.048. 23159940

[ref34] Rajagopalan S.; Sade R. S.; Townsley F. M.; Fersht A. R. Mechanistic Differences in the Transcriptional Activation of P53 by 14–3-3 Isoforms. Nucleic Acids Res. 2010, 38 (3), 893–906. 10.1093/nar/gkp1041. 19933256 PMC2817464

[ref35] Molina D. M.; Jafari R.; Ignatushchenko M.; Seki T.; Larsson E. A.; Dan C.; Sreekumar L.; Cao Y. H.; Nordlund P. Monitoring Drug Target Engagement in Cells and Tissues Using the Cellular Thermal Shift Assay. Science 2013, 341 (6141), 84–87. 10.1126/science.1233606. 23828940

[ref36] Nagasawa I.; Muroi M.; Kawatani M.; Ohishi T.; Ohba S. I.; Kawada M.; Osada H. Identification of a Small Compound Targeting PKM2-Regulated Signaling Using 2D Gel Electrophoresis-Based Proteome-wide CETSA. Cell Chem. Biol. 2020, 27 (2), 186–196 e4. 10.1016/j.chembiol.2019.11.010. 31813846

[ref37] Kayed R.; Head E.; Thompson J. L.; McIntire T. M.; Milton S. C.; Cotman C. W.; Glabe C. G. Common structure of soluble amyloid oligomers implies common mechanism of pathogenesis. Science 2003, 300 (5618), 486–489. 10.1126/science.1079469. 12702875

[ref38] Arrasate M.; Mitra S.; Schweitzer E. S.; Segal M. R.; Finkbeiner S. Inclusion body formation reduces levels of mutant huntingtin and the risk of neuronal death. Nature 2004, 431 (7010), 805–10. 10.1038/nature02998. 15483602

[ref39] Weisberg S. J.; Lyakhovetsky R.; Werdiger A. C.; Gitler A. D.; Soen Y.; Kaganovich D. Compartmentalization of Superoxide Dismutase 1 (SOD1G93A) Aggregates Determines Their Toxicity. Proc. Natl. Acad. Sci. U. S. A. 2012, 109 (39), 15811–15816. 10.1073/pnas.1205829109. 22967507 PMC3465386

[ref40] Tyedmers J.; Mogk A.; Bukau B. Cellular Strategies for Controlling Protein Aggregation. Nat. Rev. Mol. Cell Biol. 2010, 11 (11), 777–788. 10.1038/nrm2993. 20944667

[ref41] Kaganovich D.; Kopito R.; Frydman J. Misfolded Proteins Partition between Two Distinct Quality Control Compartments. Nature 2008, 454 (7208), 1088–1095. 10.1038/nature07195. 18756251 PMC2746971

[ref42] Park J. H.; Jang H. R.; Lee I. Y.; O J. K.; Choil E. J.; Rhim H.; Kang S. Amyotrophic lateral sclerosis-related mutant superoxide dismutase 1 aggregates inhibit 14–3-3-mediated cell survival by sequestration into the JUNQ compartment. Hum. Mol. Jent. 2017, 26 (18), 3615–3629. 10.1093/hmg/ddx250. 28666328

[ref43] Zhang Y.; Benmohamed R.; Zhang W.; Kim J.; Edgerly C. K.; Zhu Y.; Morimoto R. I.; Ferrante R. J.; Kirsch D. R.; Silverman R. B. Chiral Cyclohexane 1,3-Diones as Inhibitors of Mutant SOD1-Dependent Protein Aggregation for the Treatment of ALS. ACS Med. Chem. Lett. 2012, 3 (7), 584–587. 10.1021/ml3000963. 22837812 PMC3402085

[ref44] Griffin M. E.; Jensen E. H.; Mason D. E.; Jenkins C. L.; Stone S. E.; Peters E. C.; Hsieh-Wilson L. C. Comprehensive Mapping of O-GlcNAc Modification Sites Using a Chemically Cleavable Tag. Mol. Biosyst. 2016, 12 (6), 1756–1759. 10.1039/C6MB00138F. 27063346 PMC4905554

[ref45] Keller A.; Nesvizhskii A. I.; Kolker E.; Aebersold R. Empirical Statistical Model To Estimate the Accuracy of Peptide Identifications Made by MS/MS and Database Search. Anal. Chem. 2002, 74 (20), 5383–5392. 10.1021/ac025747h. 12403597

[ref46] Nesvizhskii A. I.; Keller A.; Kolker E.; Aebersold R. A Statistical Model for Identifying Proteins by Tandem Mass Spectrometry. Anal. Chem. 2003, 75 (17), 4646–4658. 10.1021/ac0341261. 14632076

