# Supplementary material for: Target Identification of a Class of Pyrazolone Protein Aggregation Inhibitor Therapeutics for Amyotrophic Lateral Sclerosis
Source: ACS Cent Sci. 2023 Dec 20;10(1):87–103. doi: 10.1021/acscentsci.3c00213 (PMC10823514; doi:10.1021/acscentsci.3c00213)
Supplement: Supplementary file 1 — oc3c00213_si_001.pdf [file oc3c00213_si_001.pdf]

Supporting Information

Target Identification for a Class of Pyrazolone Protein Aggregation Inhibitor Therapeutics for Amyotrophic Lateral Sclerosis

Pathum M. Weerawarna,[a,†], Isaac T. Schiefer,[a,††], Pedro Soares,[a] Susan Fox,[b] Richard I. Morimoto,[b] Rafael D. Melani,[c] Neil L. Kelleher,[a,b,c] Chi-Hao Luan.[d] Richard B. Silverman*[a,b,e]

[a]Department of Chemistry, Chemistry of Life Processes Institute, and Center for Developmental Therapeutics, Northwestern University, Evanston, Illinois 60208, United States

[b]Department of Molecular Biosciences, Northwestern University, Evanston, Illinois, 60208, United States

[c]Department of Chemistry and Proteomics Center of Excellence, Northwestern University, Evanston, Illinois 60208, United States

[d]High Throughput Analysis Laboratory, Chemistry of Life Processes Institute, and Department of Molecular Biosciences, Northwestern University, Evanston, Illinois 60208, United States

[e]Department of Pharmacology, Feinberg School of Medicine, Northwestern University, Chicago, Illinois 60611, U.S.A.

[†]Current address: Division of Clinical Pharmacology, Indiana University School of Medicine, Indianapolis, Indiana 46202, United States

[††]Current address: Department of Medicinal and Biological Chemistry and Center for Drug Design and Development, College of Pharmacy and Pharmaceutical Sciences, University of Toledo, Toledo, Ohio, 43614, United States

**To whom correspondence should be addressed: email: r-silverman@northwestern.edu*

Table of Content

- Visualization protocol for covalently modified proteins ………………………...……S2
    - Figure S1 ………………………………………………S2
- Full-gel image corresponding to Figure 3B in the main text ………………..…...…..…..S3
    - Figure S2 ………………………………………………S3
- Effect of MG-132 on 25 kDa covalently modified band intensity ……………………..S4
    - Figure S3 ………………………………………………S4
- Competition studies of PRAPP ……………………………………………..…..S5-S7
    - Figure S4 ………………………………………………S5
    - Figure S5 ………………………………………………S6
    - Figure S6 ………………………………………………S7
- Fluorescent-guided gel excision ……………………………………………………..S8
    - Figure S7 ………………………………………………S8
- FTS studies of PRAPP, P_2 and P_1 with 14-3-3 isoforms ………………………….S8-S9
    - Figure S8 ………………………………………………S8
    - Figure S9 ……………………………………………….....S9
    - Figure S10 ……………………………………………....…S9
- 1H NMR Spectra and MS traces …………………………………..…S10-S16
- Table S1. Proteomics analysis results ……………………………………...S17

1. Visualization protocol for covalently modified proteins

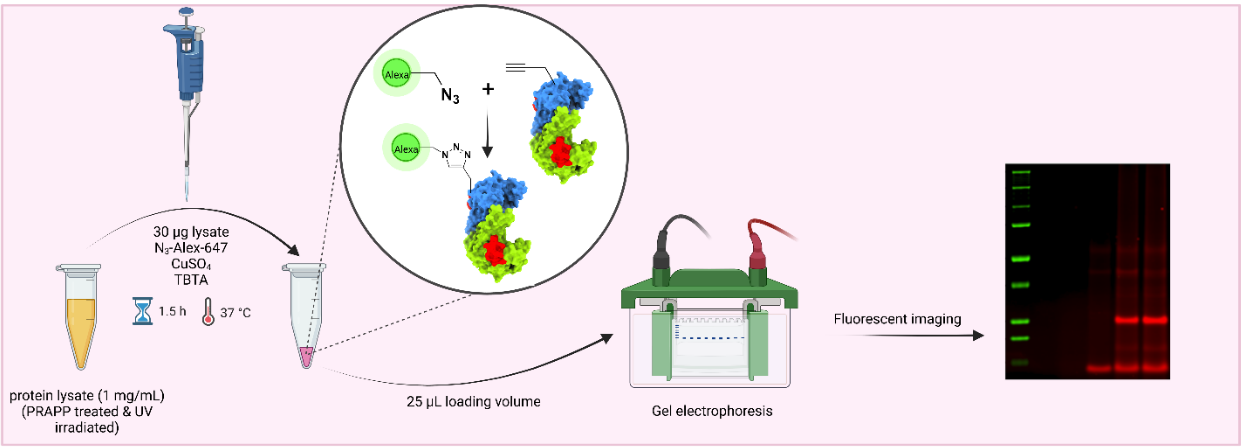

Figure S1. Visualization protocol for covalently modified proteins using N_3-Alexa-647

2. Full-gel image corresponding to Figure 3B in the main text

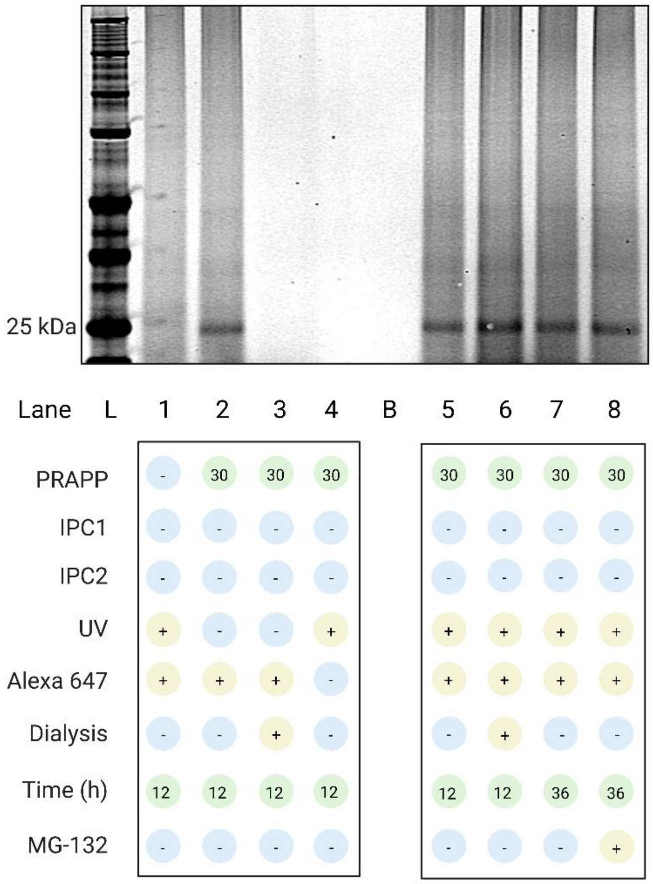

Figure S2: Full-Gel Image Corresponding to Treatment Scenarios 1, 2, and 3. (L = Ladder Lane, B = Blank Lane; the blank lane is used to separate the Alexa 647 (+), UV (+) condition from the Alexa 647 (-), UV (+) condition.)

3. Effect of MG-132 on 25 kDa covalently modified band intensity

A

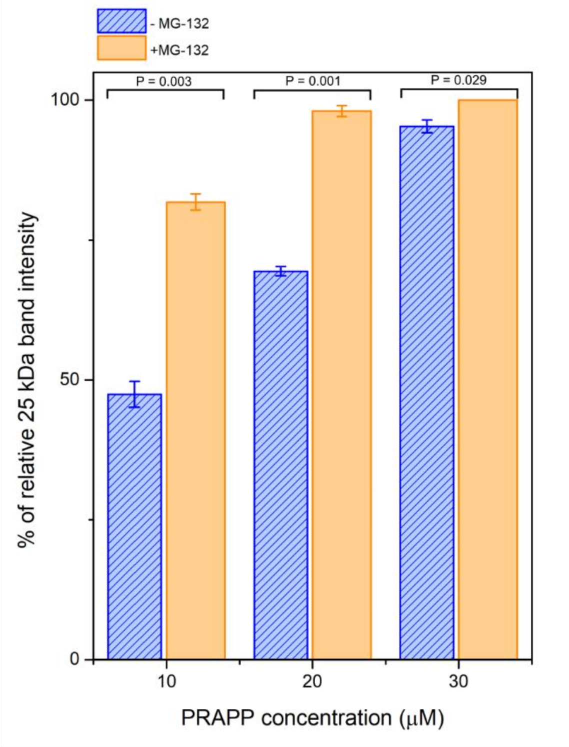

25 kDa

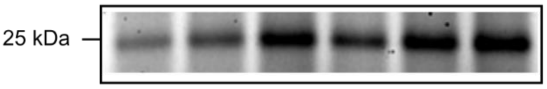

PRAPP (uM)	10	20	30	10	20	30
Time	36h	36h	36h	36h	36h	36h
MG-132 (100 nM)	-	-	-	+	+	+

B

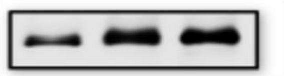

PRAPP (uM)	20	20	20
MG-132 (uM)	1	10	20

Figure S3. (A) Comparison of the 25 kDa band intensities with or without MG-132 (p values of the ANOVA included). A significant intensity increase of the 25 kDa band was observed at each concentration of PRAPP in the presence of MG-132. The difference was prominent at lower concentrations of PRAPP. **(B)** MG-132 dependant increase of the 25 kDa band intensity.

4. Competition studies of PRAPP

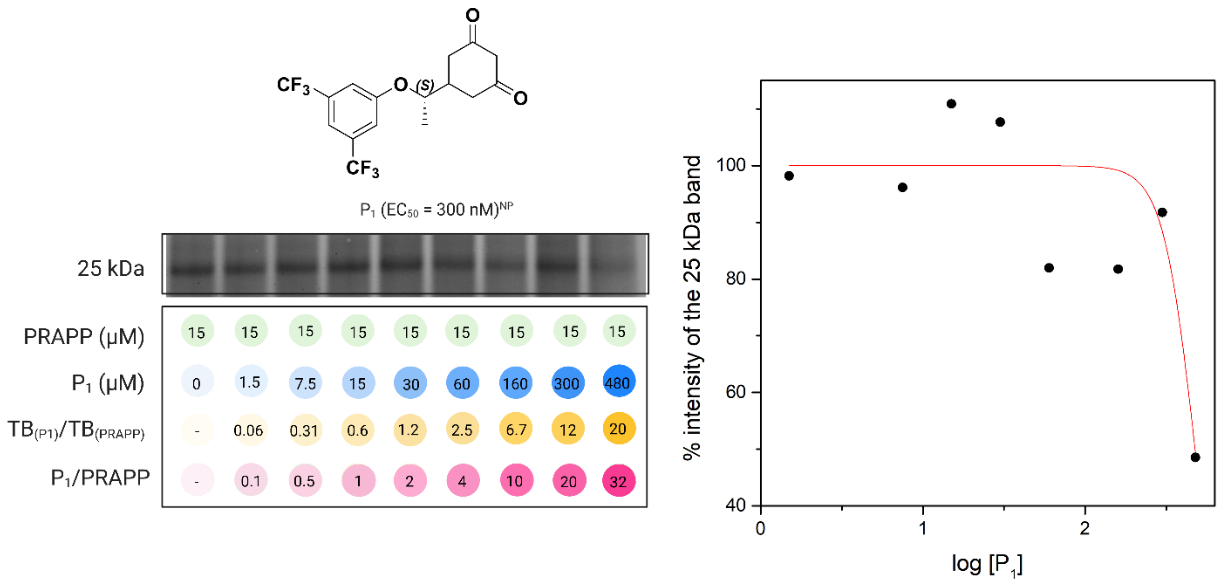

Figure S4. In-gel fluorescence of the competition studies of the PRAPP with P_1 ($TB_{(P1)}/TB_{(PRAPP)}$ and $TB_{(P1)}/TB_{(PRAPP)}$ are the ratios between the target-competitor and target-PRAPP; n = 2). P_1 did not compete out the 25 kDa covalently modified band in a concentration-dependent manner. (There is a slight reduction of the 25 kDa band intensity at 480 μM P_1 concentration)

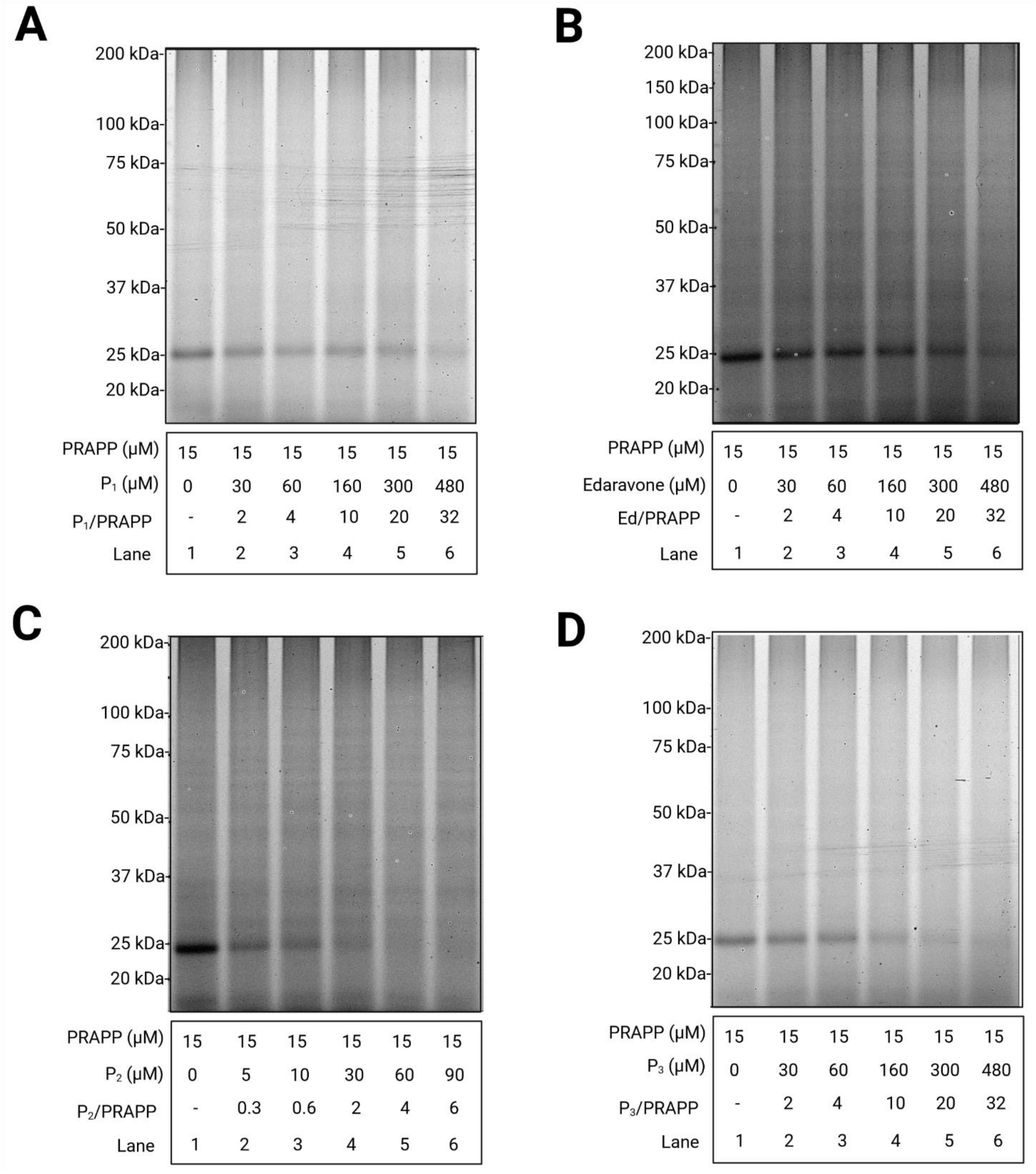

Figure S5. In-gel fluorescence of the initial 5-dose competition studies of the PRAPP in PC12-SOD1^{G93A} cells with **(A)** P_1 and **(B)** Edaravone **(C)** P_2, and **(D)** P_3. Full gel images are given to demonstrate the degree of background modifications.

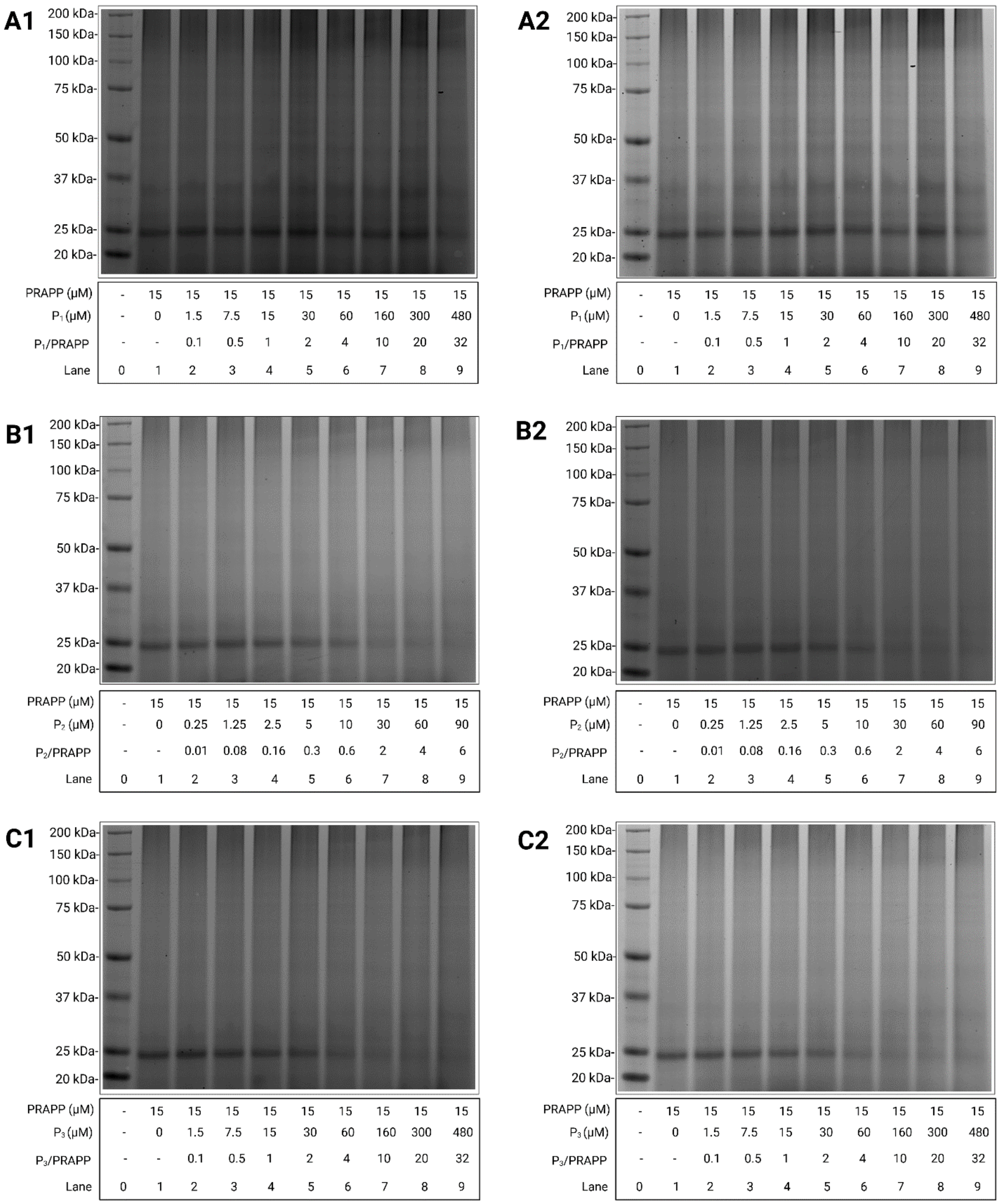

Figure S6. In-gel fluorescence of the final 8-dose competition studies of the PRAPP in PC12-SOD1^{G93A} cells with **(A1-A2)** P_1 and **(B1-B2)** P_2, and **(C1-C2)** P_3. Full gel images are given to demonstrate the degree of background modifications. (n=2)

5. Fluorescent-guided gel excision

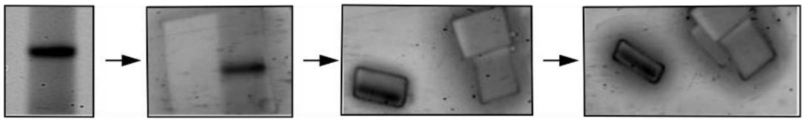

Figure S7. Fluorescence-guided gel band excision.

6. FTS sudies of PRAPP, P_2 and P_1 with 14-3-3 isoforms

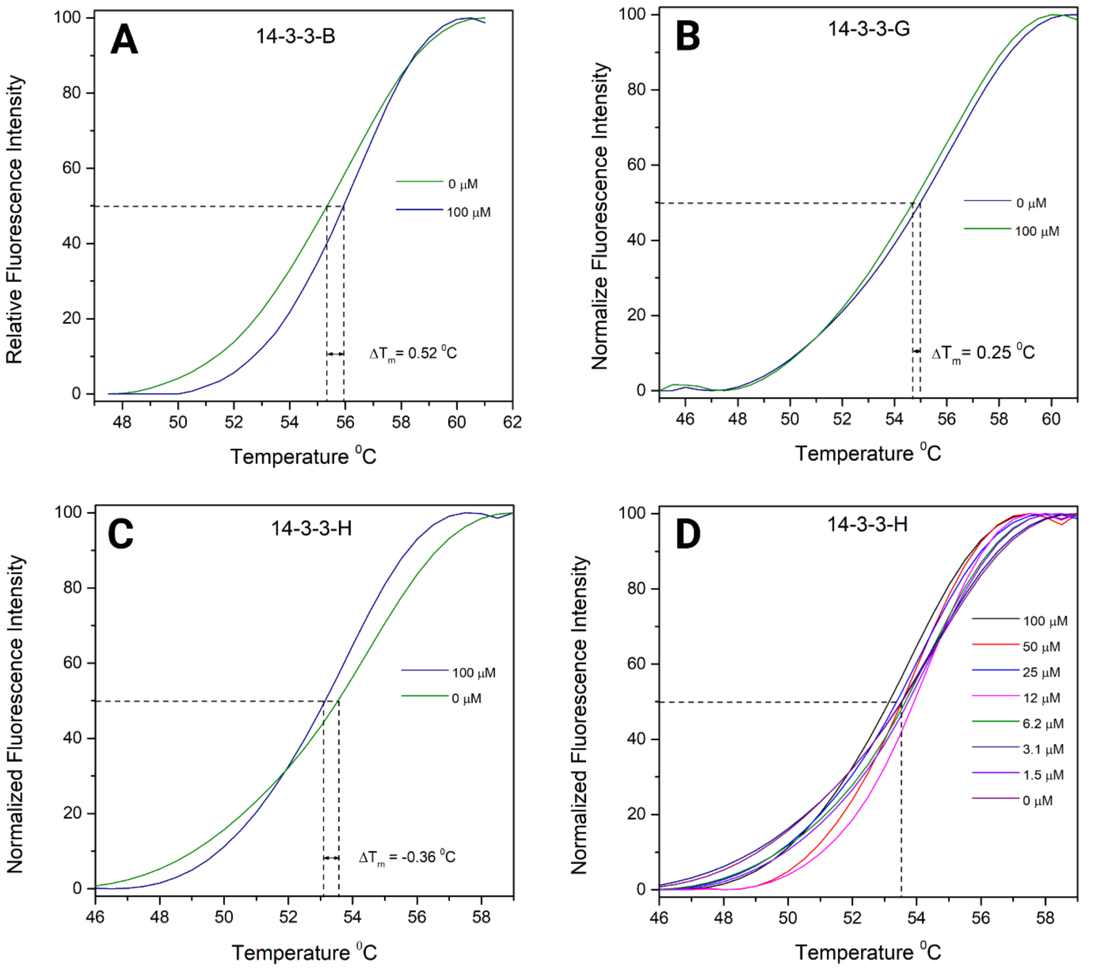

Figure S8. FTS experiment results of P_2 with **(A)** 14-3-3-B **(B)** 14-3-3-G, and **(C)** 14-3-3-H at 0 and 100 μM P_2 concentrations. (D) FTS experiment results of P_2 with 14-3-3-H at different P_2 concentrations.

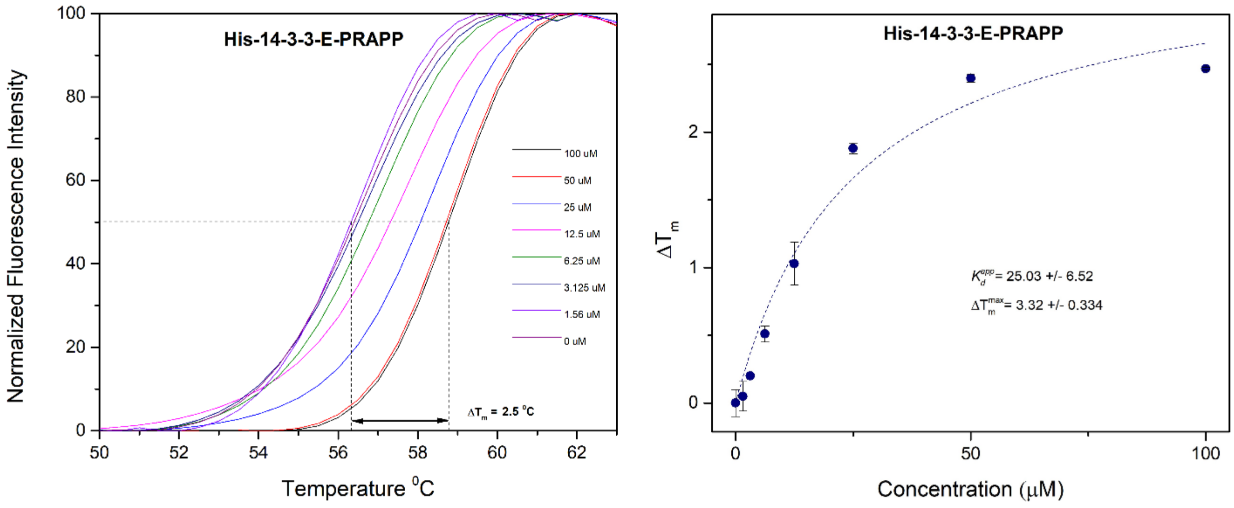

Figure S9. FTS experiment results of PRAPP with His-14-3-3-E (Melting temperature of the His-tag 14-3-3-E (~56 ºC) has shifted 2 ºC compared to His-tag free 14-3-3-E (~54 ºC)). Melting curves are shown in the left panel, and the ΔT_m vs PRAPP concentration fitting curves are shown in the right panel. (n = 2)

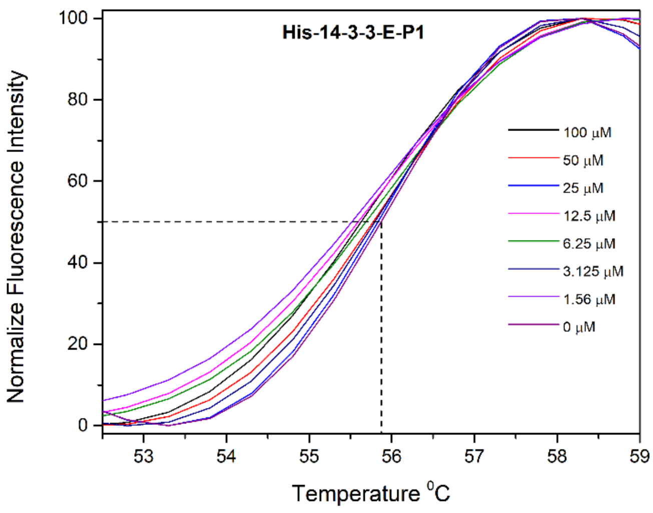

Figure S10. FTSA experiment results of P_1 with His-14-3-3-E (No Tm shift was observed with increasing concentration of P_1, indicating lack of binding).

7. ^{1}H NMR spectra and MS traces

N-(4-Azido-2,3,5,6-tetrafluorobenzyl)prop-2-yn-1-amine (9) (IPC2) (PW-II-08).

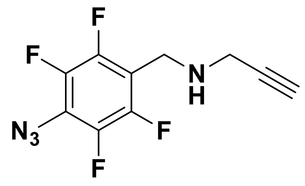

9

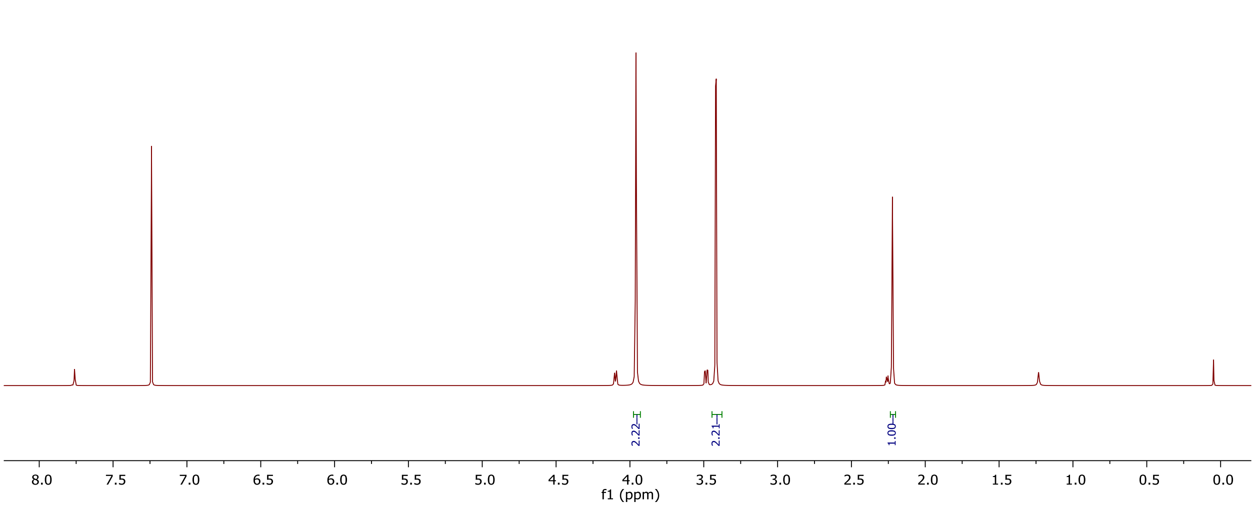

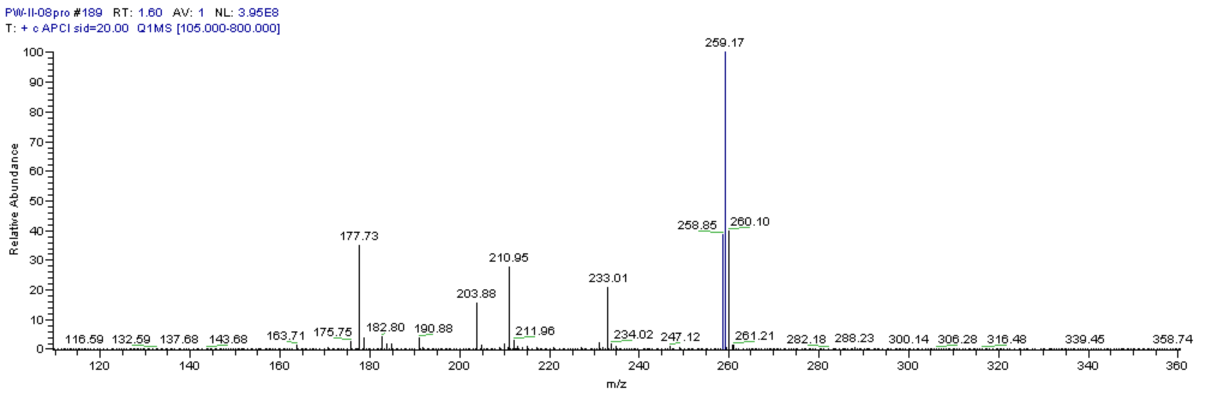

Ethyl 4-((4-methoxybenzyl)oxy)-3-oxobutanoate (10) (PW-I-167).

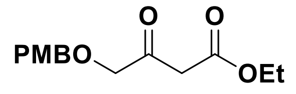

10

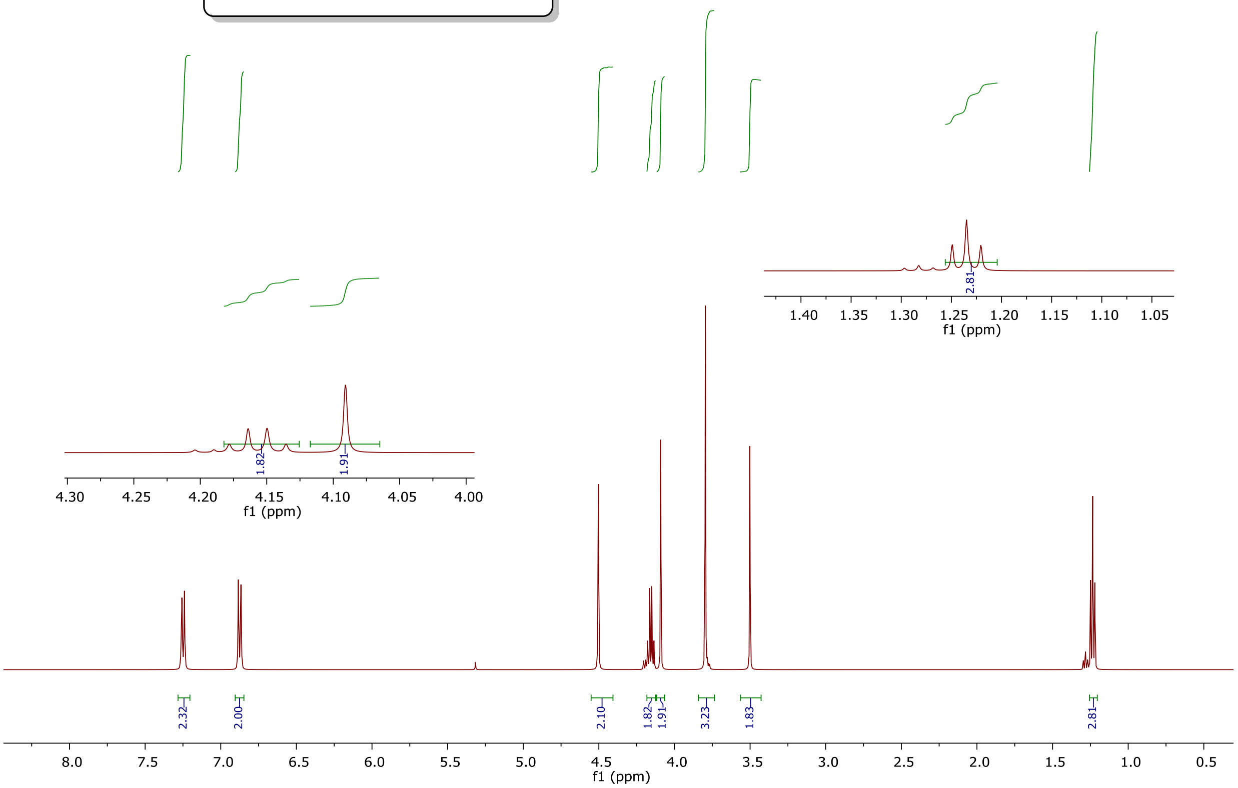

5-(((4-Methoxybenzyl)oxy)methyl)-1,2-dihydro-3H-pyrazol-3-one (11) (PW-I-176).

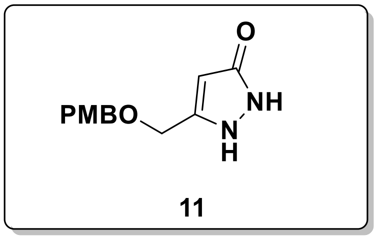

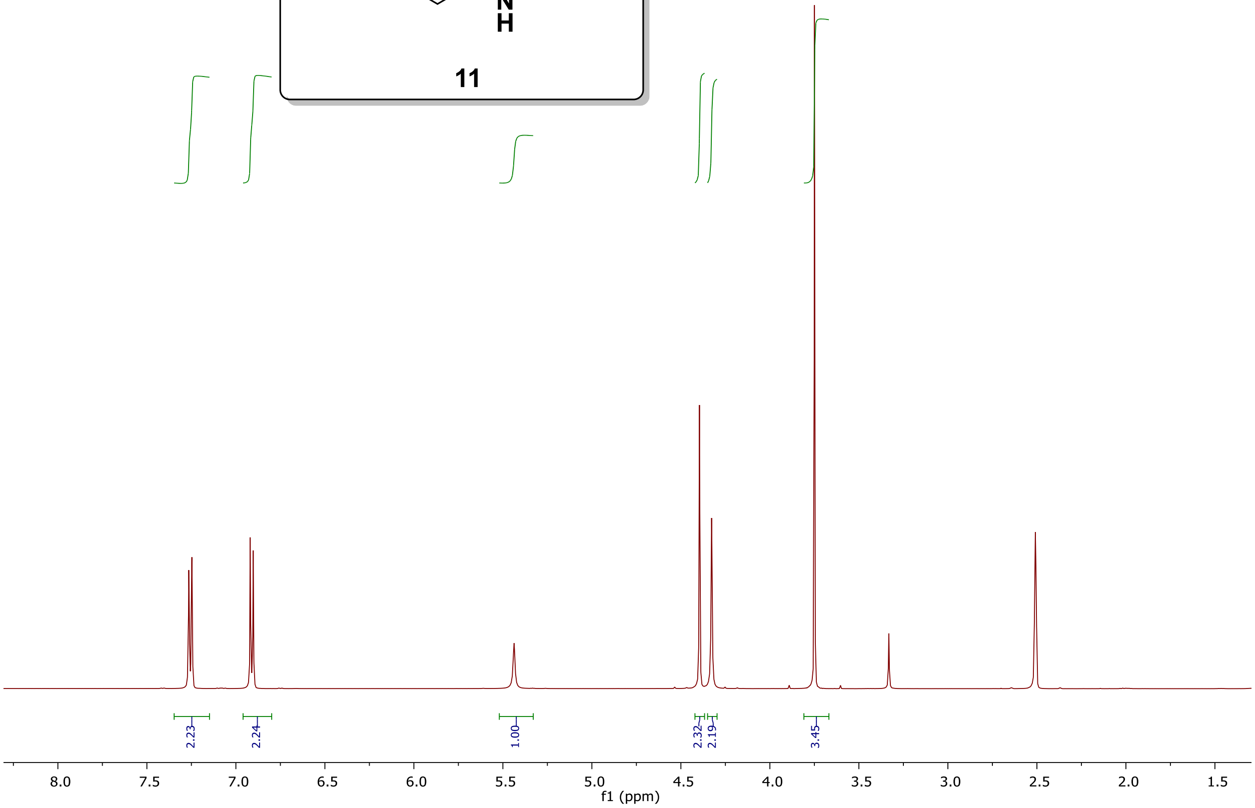

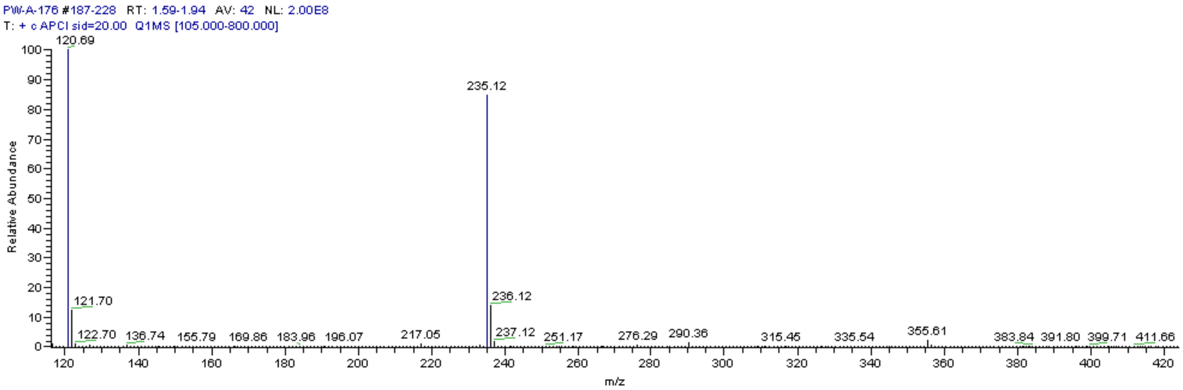

1,1'-(5-(((4-Methoxybenzyl)oxy)methyl)-3-oxo-1H-pyrazole-1,2(3H)-diyl)bis(ethan-1-one) (12) (PW-I-177).

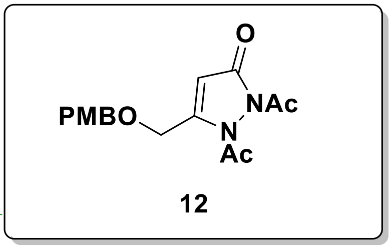

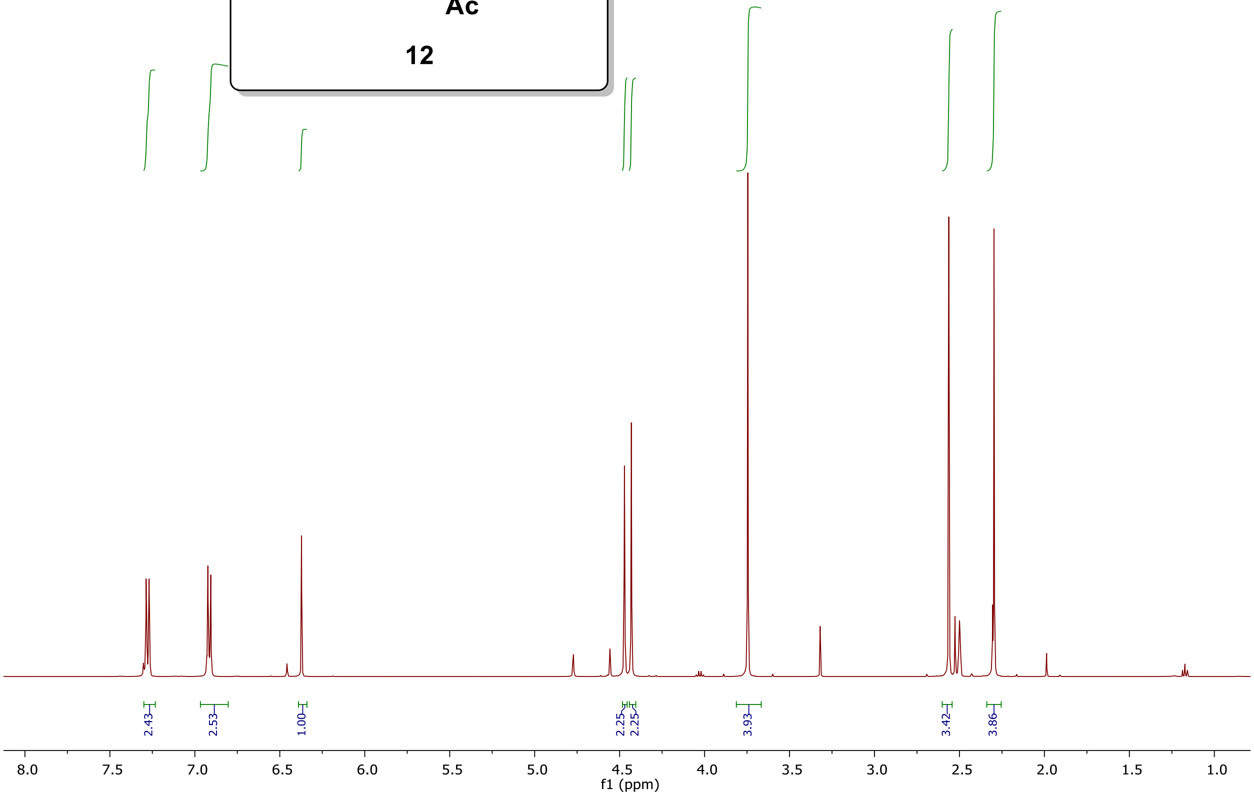

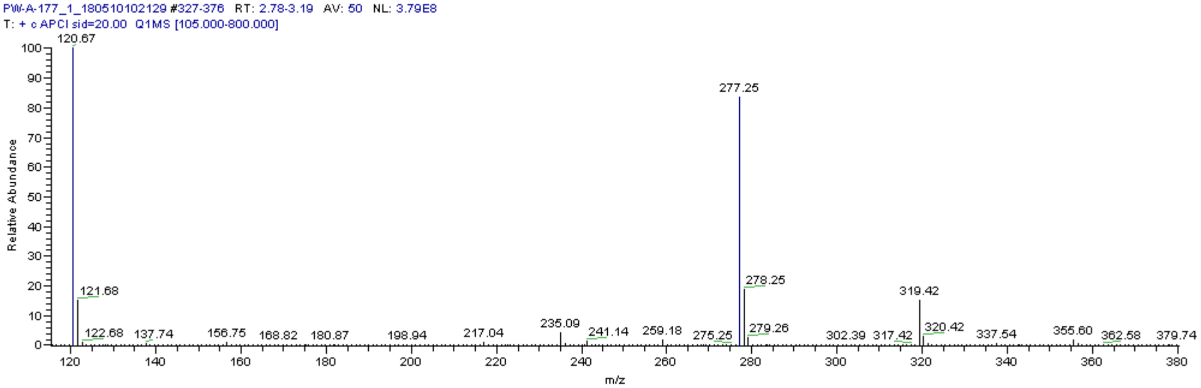

1,1'-(5-(Hydroxymethyl)-3-oxo-1H-pyrazole-1,2(3H)-diyl)bis(ethan-1-one) (13) (PW-II-137)/(PW-II-09).

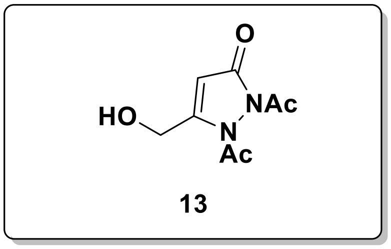

13

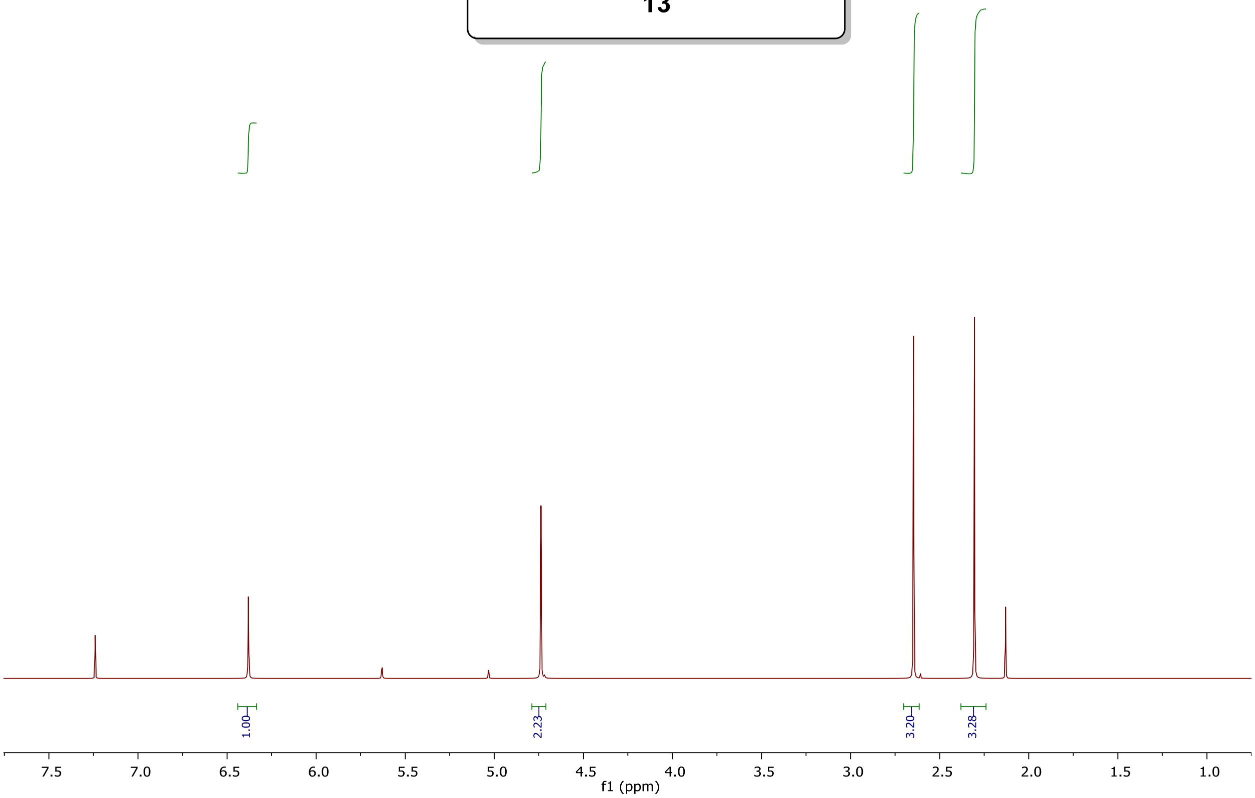

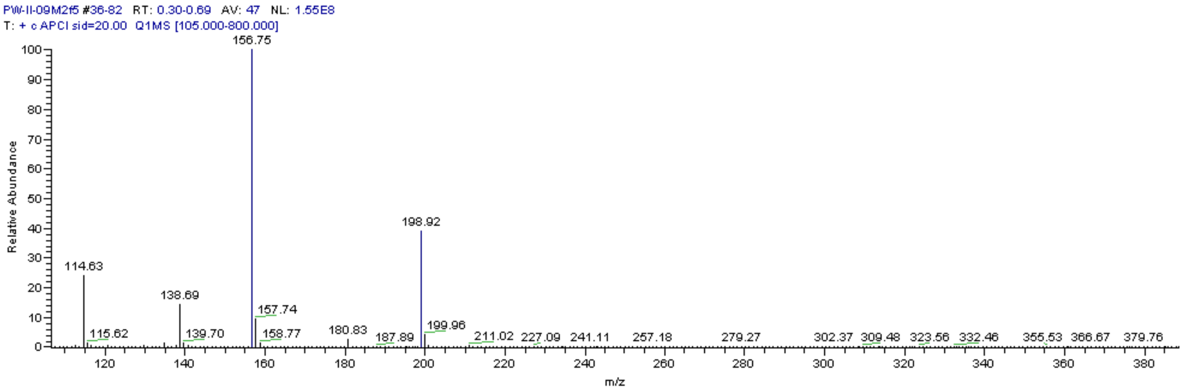

1,1'-(5-(Bromomethyl)-3-oxo-1H-pyrazole-1,2(3H)-diyl)bis(ethan-1-one) (14) (PW-II-138).

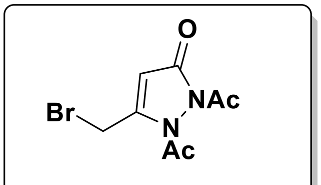

14

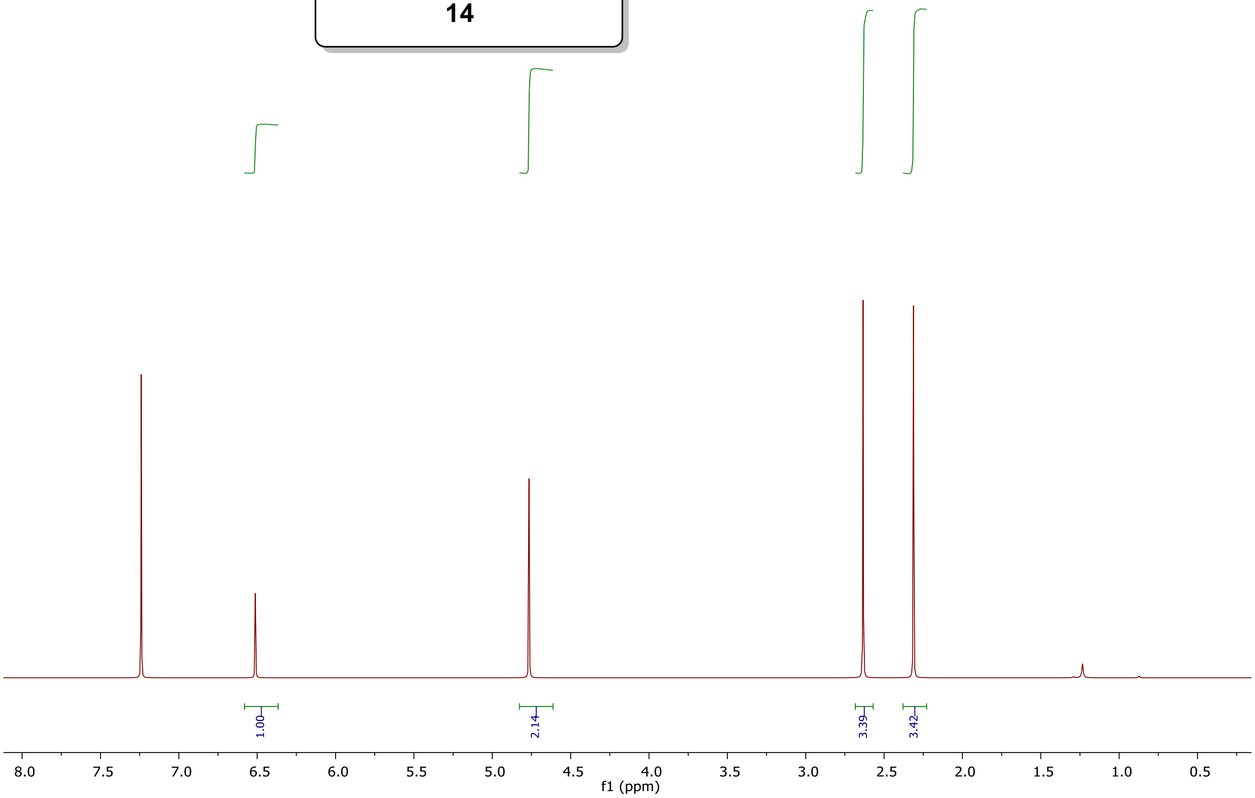

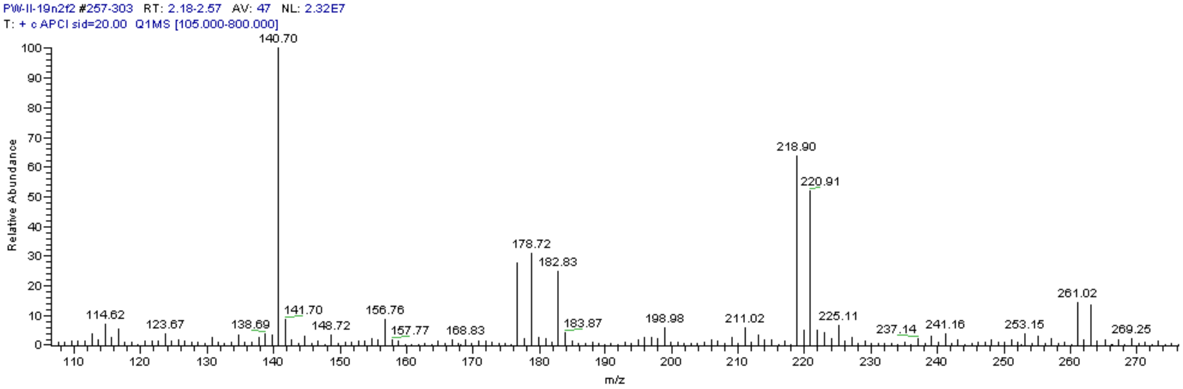

5-(((4-Azido-2,3,5,6-tetrafluorobenzyl)(prop-2-yn-1-yl)amino)methyl)-1,2-dihydro-3H-pyrazol-3-one (16) (PRAPP). (PW-II-27)

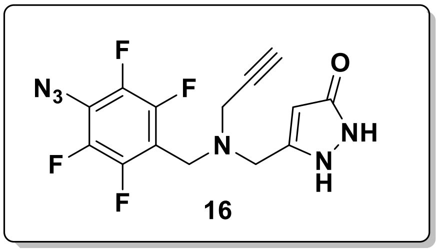

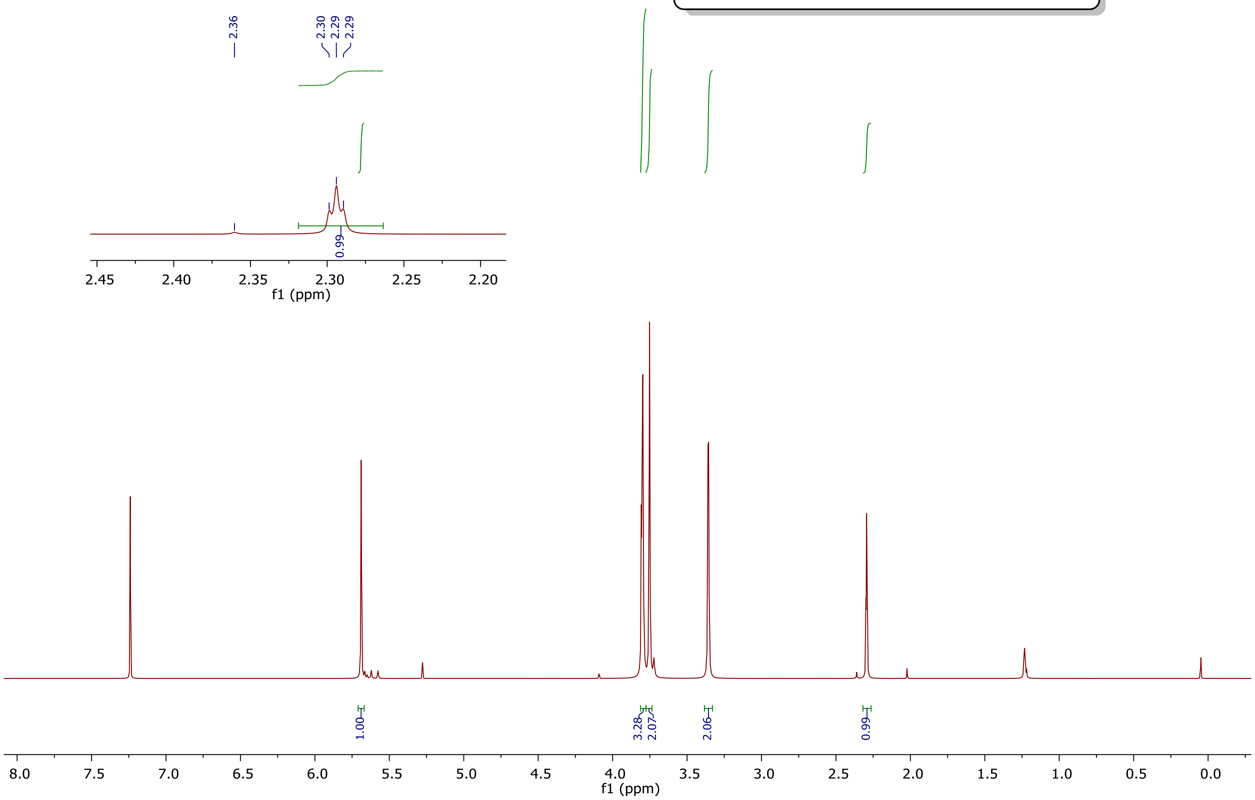

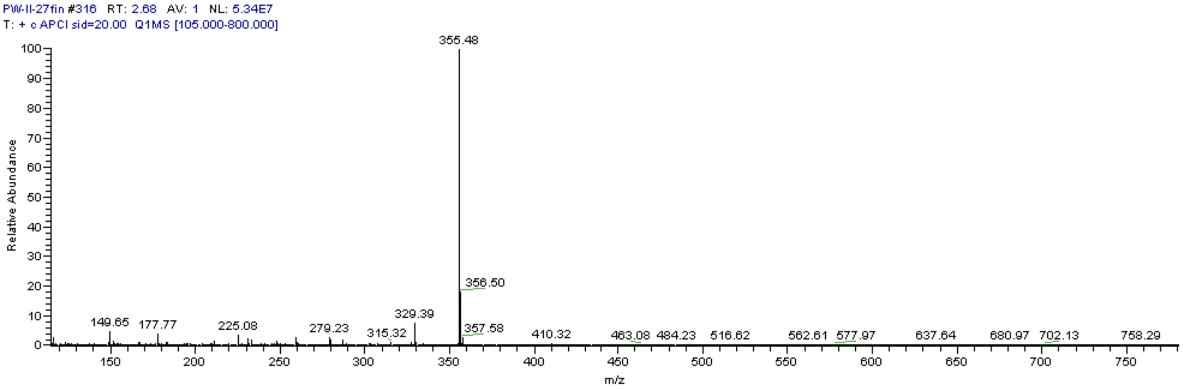

Table S1. Proteomics Analysis Results

#	Protein Name	MW (kDa)	Normalized total spectra		% Coverage		Protein ID probability %	
			control	Pull-down	control	Pull-down	control	Pull-down
1	14-3-3 protein beta/alpha OS=Rattus norvegicus OX=10116 GN=Ywhab	28	4	13	3.25	14.60	8	100
2	14-3-3 protein epsilon OS=Rattus norvegicus OX=10116 GN=Ywhae	29	ND	1	ND	7.45	ND	99
3	14-3-3 protein eta OS=Rattus norvegicus OX=10116 GN=Ywhah	28	4	17	3.25	21.10	27	100
4	14-3-3 protein gamma OS=Rattus norvegicus OX=10116 GN=Ywhag	28	4	12	3.24	14.20	16	100
5	14-3-3 protein theta OS=Rattus norvegicus OX=10116 GN=Ywhaq	28	4	25	3.27	17.10	32	100
6	Heat shock 27kDa protein 1 OS=Rattus norvegicus OX=10116 GN=Hspb1	23	ND	16	ND	16.00	ND	100
7	RAB5C, member RAS oncogene family OS=Rattus norvegicus	23	0	9	ND	11	ND	100
8	Glutathione S-transferase Mu 1 OS=Rattus norvegicus OX=10116	26	0	7	ND	15	ND	100
9	Triosephosphate isomerase OS=Rattus norvegicus OX=10116	27	2	3	4	14	100	100
10	Ras-related protein Rab-11B OS=Rattus norvegicus OX=10116	24	0	1	ND	8.8	ND	100
